# Productive and physiological implications of top-dress addition of branched-chain amino acids and arginine on lactating sows and offspring

**DOI:** 10.1186/s40104-022-00819-8

**Published:** 2023-03-07

**Authors:** Diana Luise, Federico Correa, Claudio Stefanelli, Aude Simongiovanni, Tristan Chalvon-Demersay, Maddalena Zini, Luciano Fusco, Paolo Bosi, Paolo Trevisi

**Affiliations:** 1 Department of Agricultural and Food Sciences, University of Bologna, 40127 Bologna, Italy; 2 Department for Life Quality Studies, Alma Mater Studiorum, University of Bologna, 47921 Rimini, Italy; 3 Metex Noovistago, 32 Rue Guersant, 75017 Paris, France; 4 Department of Biomedical and Neuromotor Sciences, Alma Mater Studiorum, University of Bologna, 40126 Bologna, Italy; 5 Freelancer, Reggio nell’Emilia, Italy

**Keywords:** Microbiota, Post-weaning mortality, Prolactin, Sows diet

## Abstract

**Background:**

Branched-chain amino acids (BCAAs), including *L-*leucine (*L*-Leu), *L*-isoleucine (*L*-Ile), *L-*valine (*L*-Val), and *L-*arginine (*L*-Arg), play a crucial role in mammary gland development, secretion of milk and regulation of the catabolic state and immune response of lactating sows. Furthermore, it has recently been suggested that free amino acids (AAs) can also act as microbial modulators. This study aimed at evaluating whether the supplementation of lactating sows with BCAAs (9, 4.5 and 9 g/d/sow of *L-*Val, *L*-Ile and *L*-Leu, respectively) and/or *L*-Arg (22.5 g/d/sow), above the estimated nutritional requirement, could influence the physiological and immunological parameters, microbial profile, colostrum and milk composition and performance of sows and their offspring.

**Results:**

At d 41, piglets born from the sows supplemented with the AAs were heavier (*P* = 0.03). The BCAAs increased glucose and prolactin (*P* < 0.05) in the sows’ serum at d 27, tended to increase immunoglobulin A (IgA) and IgM in the colostrum (*P* = 0.06), increased the IgA (*P* = 0.004) in the milk at d 20 and tended to increase lymphocyte% in the sows’ blood at d 27 (*P* = 0.07). Furthermore, the BCAAs tended to reduce the Chao1 and Shannon microbial indices (*P* < 0.10) in the sows’ faeces. The BCAA group was discriminated by Prevotellaceae_UCG-004, Erysipelatoclostridiaceae UCG-004, the Rikenellaceae_RC9_gut_group and *Treponema* *berlinense*. Arginine reduced piglet mortality pre- (d 7, d 14) and post-weaning (d 41) (*P* < 0.05). Furthermore, Arg increased the IgM in the sow serum at d 10 (*P* = 0.05), glucose and prolactin (*P* < 0.05) in the sow serum at d 27 and the monocyte percentage in the piglet blood at d 27 (*P* = 0.025) and their jejunal expression of *NFKB2* (*P* = 0.035) while it reduced the expression of *GPX-2* (*P* = 0.024). The faecal microbiota of the sows in Arg group was discriminated by Bacteroidales. The combination of BCAAs and Arg tended to increase spermine at d 27 (*P* = 0.099), tended to increase the Igs (IgA and IgG, *P* < 0.10) at d 20 in the milk, favoured the faecal colonisation of Oscillospiraceae UCG-005 and improved piglet growth.

**Conclusion:**

Feeding Arg and BCAAs above the estimated requirements for milk production may be a strategy to improve sow productive performance in terms of piglet average daily gain (ADG), immune competence and survivability via modulation of the metabolism, colostrum and milk compositions and intestinal microbiota of the sows. The synergistic effect between these AAs, noticeable by the increase of Igs and spermine in the milk and in the improvement of the performance of the piglets, deserves additional investigation.

**Supplementary Information:**

The online version contains supplementary material available at 10.1186/s40104-022-00819-8

## Introduction

An adequate nutrient intake by sows during lactation is of crucial importance as it can positively influence the sow's reproductive performance and body condition, reducing their weight loss and favouring a rapid recovery from the effects of the lactation period [1]. In particular, providing an adequate amount of amino acids (AAs) for the estimated requirement of lactating sows is important to sustain the tissue growth and milk synthesis of the mammary gland (MG) [2]. Amino acid deficiency during late gestation and lactation can lead to the decreased productivity of sows and to a significant decrease in their longevity [3]. In addition to being the building blocks of protein synthesis, AAs are key functional and signalling molecules in the body which can support the health and performance of animals [4]. Of the AAs, the branched-chain amino acids (BCAAs), including leucine (Leu), isoleucine (Ile) and valine (Val), and arginine (Arg), play a crucial role in MG development, the secretion of milk and the regulation of the catabolic state of the sow [5–7]. Lactating sows have a high requirement of BCAAs as they are major components of milk proteins, and they can be catabolised to provide glutamine and glutamate in the mammary tissue [5]. The supplementation of lactating sows with increasing dietary levels of BCAAs has improved the weaning weight and weight gain of litters [5, 6], and has decreased piglet mortality [8]. The effect of increasing the level of dietary Arg above the known requirements has been more intensively studied in gestating sows while its supplementation during the lactation period has been less studied, notwithstanding the potential role of Arg. In fact, Pérez Laspiur et al. [7] and Mateo et al. [9] have demonstrated that increasing dietary Arg in lactating sows reduced the catabolic state of sows by modifying their insulin status. Arginine can stimulate the secretion of key anabolic hormones, including insulin, growth hormone (GH) and prolactin [9, 10] which can promote MG development and milk synthesis [7, 11, 12]. On the contrary, Krogh et al. [13] observed no clear beneficial effect in feeding an additional 25 g/d to lactating sows. Oksbjerg et al. [14] observed that the beneficial effect of feeding Arg during late gestation and lactation improved piglet performance only until d 14 of lactation; however, this improvement disappeared at d 21 and weaning.

The dietary supplementation of AAs can also help modulate the gut microbial community as evidenced in the recent literature [15–17]. In particular, Spring et al. [16] suggested that BCAAs could modulate the faecal microbiome in post-weaning piglets, and Luise et al. [15] reported a mild influence on the faecal microbiota of pregnant sows fed with Arg. Amino acids can be metabolised both by the host and by microbes in the gut lumen; therefore, they can play a key role in bacterial survival and the bacterial metabolism which could drive the synthesising of the active molecules involved in regulating signal transduction, the nutritional metabolism, and immunity [18]. Modulation of the gut microbiota could be important, mainly in lactating sows in which the microbiome can influence the regulation of energy use to support milk production and therefore affect the maturation and growth of the offspring [19]. Furthermore, metabolites which are derived from microbes could also participate in immune modulation [18]; previous studies have suggested that BCAAs and Arg can modulate the mucosal and systemic immune response in weaning pigs [20] and sows [13, 21]; however, little information regarding the mode of action is available.

In addition, an increase in both BCAAs and Arg in the diet could have a synergetic effect by means of activating the target of rapamycin complex (mTOR) cell signalling [5]; in fact, in rats, BCAAs can inhibit the catabolism of Arg in the mammary tissue and increase its availability [22], therefore, the increased availability of Arg would increase the synthesis of nitric oxide which acts as a major vasodilator increasing the blood flow and, in turn, the uptake of nutrients [5, 17]. However, to the authors' knowledge, no study has investigated this potential synergic effect between BCAAs and Arg. Furthermore, still little is known regarding the effect that BCAAs and Arg supplementation over the nutritional requirements may have on the intestinal microbiota and physiology of lactating sows and, as a consequence, on the modulation of the immunity and health of their offspring. Therefore, the aim of the present study was to investigate the effect of dietary supplementation with BCAAs, and Arg alone or in combination on the physiological parameters, microbial profile, colostrum and milk composition and performance of sows and their offspring during lactation and post weaning.

## Material and methods

### Ethics

The in vivo trial was conducted in a commercial multiplication unit located in the so-called ‘Italian Food Valley’. The animals enrolled in the present study were sows and piglets raised under conventional farm rearing conditions in Europe according to Dir. 120/2008 EC. The Italian Ministry of Health approved the experimental procedures of the trial with protocol n. 517/2018-PR. The sows were reared in conventional farrowing crates having a nesting area consisting of a full-floor under a warm lamp. The post-weaning housing facility had fully slatted concrete floors and automated ventilation and temperature controls. The temperature was started at 27 °C and was gradually decreased to approximately 21 °C as the pigs increased in weight and size. During the post-weaning phase, the piglets had continuous access to feed and water.

### Animals and sampling

A total of sixty-eight sows and their litters were enrolled in the study. One week before farrowing, the sows (average parity: 6.10 ± 0.18) were housed individually in gestation crates (2.12 m × 0.61 m). Four days before farrowing (d −4), the sows were individually weighed and assigned to one of the four dietary groups (17 sows per group) according to their parity and body weight (BW) as follows: 1) a control group, fed a standard lactating sow diet (CO); 2) a group fed the standard lactating sow diet plus *L*-Val, *L*-Ile and *L*-Leu at 9, 4.5 and 9 g/d/sow, respectively (BCAA); 3) a group fed the standard lactating sow diet plus 22.5 g Arg/d/sow (Arg); 4) a group fed the standard lactating sows’ diet plus *L*-Val, *L*-Ile, *L*-Leu and *L*-Arg at 9, 4.5, 9 and 22.5 g/d/sow, respectively, (BCAA + Arg).

Supplementation with AAs (BCAA = 22.5 g; Arg = 22.5 g; and BCAA + Arg = 45 g) was included in addition to the feed once a day during the morning meal. The CO diet was formulated as a standard lactating sow diet containing corn (27.5%), barley (22%), wheat bran (21%), soybean meal (13.5%), rice hulls (6%), roasted soybean (2%), dried beet pulp (1.5%), calcium carbonate (1.2%), dicalcium phosphate (0.9%), salt (0.3%), *L*-lysine-HCl (0.25%), *DL*-methionine (0.05%) and phyhtase (0.05%) in decreasing order. The diet was calculated to contain 16.5% crude protein, 4.95% crude fibre, 5.75% crude fat and 2300 kcal EN/kg. The AAs contents analysed are reported in Table 1. The diet was formulated to meet or exceed the National Research Council (NRC, 2012) [23] nutrient requirements for lactating sows. The analytical values of the basal diet were 100%, 130%, 120%, and 203% of the suggested values for Val, Ile; Leu and Arg, respectively. The on top supplementation, based on a feed intake of 6.61 [20], represented a further addition of 18%, 11%, 11%, and 33%, for Val, Ile, Leu and Arg, respectively. The study was carried out in 5 consecutive batches.

**Table 1:** Analysed composition of the standard diet

Item	% feed
Dry matter	89.24
Crude protein	16.2
Lysine	0.87
Threonine	0.57
Methionine	0.316
Cystine + cystein	0.279
Methionine + cystine	0.595
Tryptophan	0.199
Valine	0.74
Isoleucine	0.60
Leucine	1.18
Arginine	0.97
Phenylalanine	0.73
Tyrosine	0.51
Histidine	0.40
Serine	0.75
Alanine	0.76
Aspartic acid	1.34
Glutamic acid	3.08
Glycine	0.70
Proline	1.07

The sows were individually weighed at the beginning of the study (d −4), and on the day of weaning (d 27). At the same time points, the depth of muscle and backfat were measured using ultrasound (LS-1000, Tokyo Keiki Inc., Tokyo, Japan) at the P2 position (left side of the 10^th^ rib and 6 cm away from the spine of the sows) to estimate fat and muscle mobilisation. The loss of backfat and muscle depth was calculated as initial value—final value. The health status of the sows was followed daily. It was checked daily that the sows consumed the entire meal, that no principle of agalactia or fever occurred and that no aggressive behaviour towards the piglets appeared. When the animals demonstrated these symptoms, they were excluded from the study. No sow had been excluded for these reasons.

Within 24 h post farrowing, litter and individual offspring (piglet) characteristics including the number of born alive (BA) piglets, the number of stillborn (SB) piglets and the number of mummified foetuses were recorded. Cross-fostering of the piglets was carried out within 24 h after farrowing by sows in the same experimental group in order to standardise litter size, to match the sow’s rearing capacity with litter size and to ensure that all the piglets could access a functional teat. The piglets were individually weighed just after farrowing (d 0 before cross-fostering), at days 7 (d 7) and 14 (d 14) of lactation, the day of weaning (d 27) and at days 7 (d 34) and 14 (d 41) post-weaning in order to calculate the average daily gain (ADG) of the piglets and the litters. The health status of the piglets was followed over the entire period, and morbidity and mortality were recorded.

At d −4, d 10 and d 27, blood samples from 50% of the sows (32 sows, 8 sows per group, balanced for BW and parity order) were collected. Blood was collected at 2 h after the last meal via jugular venipuncture into a K3 EDTA tube (Vacutest Kima Srl, Arzergrande, PD, Italy) to analyse the haematological parameters, and into a tube containing a clot activator to obtain serum (Vacutest Kima Srl). Following the same procedure, at d 10 and d 27, blood samples were collected from 2 piglets per sow (2 piglets with an average BW from the sows from which blood was collected; 16 piglets per group per time point; 64 samples in total). An aliquot of blood was collected using a K3 EDTA (Vacutest Kima Srl) collection tube by venipuncture of the vena cava to analyse the haematological parameters. A second aliquot of blood was collected using a collection tube without an anticoagulant to obtain serum (Vacutest Kima Srl). Briefly, to obtain serum, the blood samples were incubated at room temperature for 2 h, then centrifuged at 3000 × *g* for 10 min. The serum samples were then used to quantify insulin, prolactin (of the sows), urea, glucose, and the immune parameter (IgA, IgG and IgM; of both the sows and the piglets) concentrations.

Colostrum and milk samples were collected from 32 sows (8 sows per group) at farrowing (colostrum) and at d 10 (milk d 10) and 20 (milk d 20) of lactation. Samples were collected across all of the sows’ teats as reported by Luise et al. [24]. One aliquot was kept at 4 °C for analysing the protein, fat, lactose, and urea concentrations, and the count of the somatic cells (SCC); a second aliquot was snap-frozen in liquid nitrogen for polyamine and immune parameter analysis. Some milk samples were unable to be collected, or an insufficient amount was obtained for each subsequent laboratory analysis; therefore, not all the sows were represented at each time point in the results for all milk composition parameters.

At weaning, a faecal sample was collected from 32 sows (8 sows per group) into a sterile collection tube after rectal stimulation. The samples were immediately frozen in liquid nitrogen and then stored at −80 °C until microbiota analysis.

Furthermore, at weaning, a total of 32 piglets (8 piglets per group among the piglets selected for blood analysis) were slaughtered, and gut mucosa from the distal part of the jejunum (75% of the small intestine length) was gently scraped and snap-frozen in liquid nitrogen and then preserved at − 80 °C for gene expression analysis.

### Blood analysis

#### Haematological and hormonal parameters

A total of 15 haematological parameters (*erythrocyte traits*: red blood cell count (RBC), haemoglobin (HGB), haematocrit (HCT), mean corpuscular volume (MCV), mean corpuscular haemoglobin (MCH), andmean corpuscular haemoglobin concentration (MCHC); *leukocyte traits*: white blood cell count (WBC), lymphocyte (LYMPHO), neutrophil (NEUTRO), eosinophil (EOSI), basophil (BASO), and monocyte (MONO); *platelet traits*: platelet count (PLT)) were detected using laser-empedimetric cytometry. Glucose and urea were detected colourimetrically in the serum samples following the manufacturer’s instructions of the ILab Chemistry System catalogue (Cat. No. 0018250840 for glucose and Cat. No. 0018255440 fro urea) and the ILab instrument (International Laboratory, Italy).

Concentrations of prolactin (ng/mL) and insulin (mU/L) were assessed in the serum samples of the sows using swine prolactin quantitation kits (SEA846Po Cloud Clone Corp., Katy, TX) and insulin quantitation kits (10–1200-01 Mercodia, Upsala, Sweden) according to the manufacturer’s instructions. Absorbance was set at 450 nm on the Multiskan multiplate reader (MultiskanTM FC Microplate Photometer—Thermo Fisher Scientific) and concentrations were calculated using a four-point parametric curve.

#### Immunoglobulins

Serum IgA, IgG and IgM were quantified on the samples collected from the sows and their respective piglets. The Igs concentration was analysed using an immunoglobulin enzyme-linked immunosorbent assay (ELISA) procedure following the protocol described by Bosi et al. [25]. The serum samples of sows were diluted at 1:7000 for IgA, 1:300,000 for IgG, 1:12,000 for IgM. The serum samples of the piglets were diluted at 1:3200 for IgA, 1:100,000 for IgG and 1:12,000 for IgM. The reaction was quantified spectrophotometrically at an absorbance of 405 nm using a microplate reader (Multiskan FC Microplate Photometer – Thermo Fisher Scientific). The data regarding Igs concentrations were calculated using a four-point parametric curve and were expressed as milligram per millilitre (mg/mL).

### Colostrum and milk analysis

Colostrum and milk composition in terms of total fat, total proteins, caseins, lactose, urea, dry matter and SCC were analysed in triplicate, assayed using the infrared spectroscope Milkoscan FT2 (FOSS A/S, Padua, Italy).

The concentrations of IgA, IgG and IgM were quantified using an immunoglobulin ELISA procedure following the protocol described by Luise et al. [26]. For the analysis, the colostrum samples were diluted at 40,000, 500,000 and 10,000 for IgA, IgG and IgM, respectively, and the milk samples were diluted at 20,000, 2400 and 4000 for IgA, IgG and IgM, respectively. The reaction was quantified spectrophotometrically at an absorbance of 405 nm using a microplate reader (Multiskan FC Microplate Photometer – Thermo Fisher Scientific). The data regarding the Igs concentrations were calculated using a four-point parametric curve and were expressed as milligram per millilitre (mg/mL).

The concentration of insulin growth factors (IGF-1) was assayed using swine IGF-1 ELISA Quantitation Kits (SEA050Po Cloud Clone, Wuhan, China) according to the manufacturer’s instructions. The reaction was quantified spectrophotometrically at an absorbance of 450 nm using a microplate reader (Multiskan FC Microplate Photometer – Thermo Fisher Scientific). The concentrations of IGF-1 were calculated using a four-point parametric curve and were expressed as µg/mL.

The concentrations of putrescine, spermidine and spermine (nmol/mL) in the colostrum and milk were assessed using high-performance liquid chromatography and were quantified using fluorimetry, according to the method described by Pinna et al. [27].

### Microbiota analysis

Total bacterial DNA for microbiota analysis was extracted from the faecal samples using the FastDNA^TM^Spin Kit for Soil, MP Biomedicals Europe, (LLC); DNA quantity and quality were evaluated using a Nanodrop ND 1000 spectrophotometer (Nanodrop Technologies Inc., Wilmington, DE, USA). The DNA was amplified for the V3-V4 hypervariable regions of the 16S rRNA gene. Amplicons were produced using the primersPro341F: 50-TCGTCGGCAGCGTCAGATGTGTATAAGAGACAGCCTACGGGNBGCASCAG-30 and Pro805R: 50GTCTCGTGGGCTCGGAGATGTGTATAAGAGACAGGACTACNVGGGTATCTAATCC-30, using PlatinumTM Taq DNA Polymerase High Fidelity (Thermo Fisher Scientific, Italy). The libraries were prepared using the standard protocol for MiSeq Reagent Kit v3 and were sequenced on the MiSeq platform (Illumina Inc., San Diego, CA, USA).

### Gene expression analysis in the jejunal mucosa

Total RNA was extracted from the piglet jejunal mucosal samples using the GeneJET RNA Purification Kit (Thermo Fisher Scientific, Waltham, MA, USA) according to the manufacturer’s instructions. DNase treatment was performed to remove contaminating DNA using the TURBO DNA-free™ DNA Removal Kit (Thermo Fisher Scientific, Waltham, MA, USA) following the recommended protocol. The quantity and quality of the RNA were evaluated using a Nanodrop ND 1000 spectrophotometer (Nanodrop Technologies Inc., Wilmington, DE, USA) and agarose gel electrophoresis, respectively. A total of 1000 ng of RNA was then converted into complementary DNA using the High-Capacity RNA-to-cDNA™ Kit (Thermo Fisher Scientific, Waltham, MA, USA) according to the manufacturer’s instructions. Duplex Real Time PCR reactions contained 2 µL cDNA and 8 µL mix containing primers, probe (Additional file 1: Table S1) and 2X TaqMan Mastermix, and were run in triplicate on the Applied Biosystems QuantStudio™ 7 Flex Real-Time PCR system (Thermo Fisher Scientific, Waltham, MA, USA) with the following thermocycler settings: 50 °C for 2 min, 95 °C for 2 min and 40 cycles of 95 °C for 1 s and 60 °C for 20 s. Hydroxymethylbilane synthase (*HMBS*) was used as housekeeping gene. The following genes were selected and analysed: innate immune signal transduction adaptor (*MyD88*), nuclear factor kappa B subunit 2 (*NFKB2*), Occludin (*OCLN*), Tight junction protein 1 (*ZO-1*), Mucin 13 cell surface associated (*MUC13*), glutathione peroxidase 2 (*GPX-*2), Claudin-4 (*CLAUD4*), Claudin-3 (*CLAUD3),* polymeric immunoglobulin receptor (*PIGR*), branched chain amino acid transaminase 2 (*BCAT2*), ornithine decarboxylase 1 (*ODC1*), solute carrier family 6 (neutral amino acid transporter), member 19 (*SLC6A19*), solute carrier family 7 member 9 (*SLC7A9),* solute carrier family 1 member 5 (*SLC1A5),* solute carrier family 38 member 2 (*SLC38A2)*.

QuantStudio Design and Analysis Software v2.5 (Thermo Fisher Scientific, Waltham, MA, USA) was used for determining the gene expression cycle threshold (Ct) values. For each sample the Ct value of the *HMBS* gene was subtracted from the Ct value of the target gene (ΔCt). The average ΔCt value of the reference animals was then subtracted from the ΔCt value of all the samples (ΔΔCt). The expression of the target gene was given as fold change calculated by 2^−ΔΔ^ ^Ct^.

### Statistical and bioinformatic analysis

Statistical analysis was carried out using SAS version 9.3 (SAS Inst. Inc., Cary, NC, USA) and R software (V 3.6).

The 4 dietary groups were considered to be arranged in a factorial manner with a 2 × 2 design, considering the two groups which were supplemented with these AAs (BCAA and BCAA + Arg) for the BCAA positive effect and, equally, the two groups which were supplemented with these AAs (Arg and BCAA + Arg) for the Arg positive effect. The CO diet was considered negative for both BCAA and Arg factors. The sows were also classified by parity as follows: 1 = parity 2 to 4, 2 = parity 5 to 7 and 3 = parity 8 to 10. A general linear model (GLM) procedure was used to fit measurements carried out on sows with a linear model including batch, class of parity order, BCAA and Arg supplementation, and their interaction, as factors. Depending on the parameters, certain covariates were also included in the model; the covariates included will be discussed in the presentation of the results. Regarding the growth performance of the piglets before and after weaning, and for piglet blood parameters, the sow was included as a random factor and a MIXED procedure was applied; in this case, the model was obtained fitting the values using Restricted Maximum Likelihood (REML) estimation; the degrees of freedom were estimated using the Kenward-Rogers function. The coefficient of variability (CV: standard deviation/mean) of the piglet weight at weaning was calculated within each litter and these values were considered to be approximated to a beta distribution [28] and analysed by the GLIMMIX procedure regarding the BCAA and Arg effects. The GENMOD procedure was used with a binomial distribution and a Logit link function was used for mortality rates in a model which included the effects of litter size at birth, batch, class of parity, BCAAs, Arg and their interaction.

In addition, the following contrasts were carried out to assess the effect of BCAA and Arg supplementation: CO vs. Addition: (CO vs. BCAA, Arg and BCAA + Arg groups), alone vs. mixed addition (BCAA and Arg groups vs. BCAA + Arg group), BCAAs alone vs. Arg alone (BCAA vs. Arg group).

The data are expressed as least-square means and standard error of the mean (SEM). A difference was declared at *P* < 0.05 and *P* > 0.05, and < 0.10 was considered a tendency.

Microbiota analysis was carried out using the DADA2 pipeline [29], and taxonomic categories were assigned using the Silva Database (release 138) as a reference [30]. Alpha (Shannon, Chao1 and Simpson indices) and Beta (calculated as Bray Curtis distance matrix) diversity, as well as the abundance of taxonomic categories, were analysed with R software 3.6, using the PhyloSeq [31], Vegan [32], lme4 [33] and MixOmics [34] packages. The alpha diversity indices were analysed using an ANOVA model including batch, class of parity order, BCAA and Arg supplementation, and their interaction, as factors. The beta diversity was analysed using a PERMANOVA model (‘Adonis’ procedure) including batch, class of parity order, BCAA and Arg supplementation and their interaction as factors. The effect of BCAAs, Arg and parity class on Bray Curtis distance was visualised using a Non-Metric Multidimensional Scaling (NMDS) approach. The difference in the taxa abundancy of the Arg, BCAA and Arg + BCAA groups as compared with the CO group was analysed using the DESeq2 package, based on negative binomial generalised linear models and applying the Benjamini–Hochberg method for multiple testing correction [35]. Furthermore, to identify the discriminant taxa of each group, the multivariate sparse partial least squares discriminant analysis (sPLS-DA) supervised approach was carried out on the microbial data at the amplicon sequence variant (ASV) level [36] following the procedure and conditions described by Luise et al. [37]. The ASVs showing a correlation of > 0.3 with the dietary group and a stability of ≥ 55% were considered discriminative.

## Results

### The effect of dietary supplementation with branched-chain amino acids and/or arginine on sow body weight, backfat and muscle depth

The sows which received Arg supplementation tended to be lighter at the end of the lactation period (*P* = 0.096; however, this was in the presence of a trend of interaction (*P* = 0.078) between the BCAAs and Arg. The orthogonal contrasts carried out among the groups showed no statistical difference in the BW of the sows at d 27. Neither the BCAA nor the Arg supplementation influenced ADG, backfat, muscle loss and the backfat loss /muscle loss ratio of the sows (Table 2). Both backfat and muscle depth loss were influenced by their respective initial depth (*P* < 0.001) and by the number of weaned piglets (coefficient for backfat: 0.252; coefficient for muscle: 0.697; *P* < 0.05); muscle loss was influenced by parity class (*P* = 0.001). The average piglet BW at d 27 significantly influenced the backfat loss (coefficient: 0.00045; *P* = 0.03) but not the muscle depth loss of the sows (Table 2).

**Table 2:** The effect of BCAA and Arg supplementation on the sow lactating diet regarding the sow’s performance traits

Item	Diet^a^				SEM	*P*-value		
	**CO**	**Arg**	**BCAA**	**BCAA + Arg**		**BCAA**	**Arg**	**BCAA × Arg**
Sows (*n*)	17	17	17	17				
Sow BW d −4, kg	302	302	301	292	7.89	0.925	0.977	0.584
Sow BW d 27, kg^b^	247	239	245	249	3.50	0.643	0.096	0.078
Sow ADG, kg^c^	−1.73	−1.95	−1.8	−1.62	0.14	0.741	0.280	0.171
Backfat loss, mm^d^	3.63	3.79	4.1	3.94	0.30	0.241	0.702	0.598
Muscle loss, mm^e^	5.82	7.11	5.27	8.69	0.85	0.642	0.203	0.193
Backfat loss/muscle loss^f^	0.65	0.73	0.17	0.78	0.34	0.301	0.879	0.447

^a^Diet: CO = the group fed a standard lactating sow diet; Arg = the group fed the standard lactating sow diet plus 22.5 g/d/sow of *L*-Arg; BCAA = the group fed the standard lactating sow diet plus *L*-Val, *L-*Ile and *L*-Leu at 9, 4.5 and 9 g/d/sow; BCAA + Arg = the group fed the standard lactating sow diet plus *L*-Val, *L*-Ile and *L*-Leu and *L*-Arg at 9, 4.5, 9 and 22.5 g/d/sow. Parity: 1 = parity 2 to 4, 2 = parity 5 to 7 and 3 = parity 8 to 10

^b^Sow BW d 27: Orthogonal contrast were not significant; parity class was significant: 1 vs. 2, *P* = 0.053; 2 vs. 3, *P* = 0.0089; 1 vs. 3, *P* < 0.0001; sow BW d −4 was included as covariate and was significant (*P* < 0.0001; coefficient = 0.590)

^c^Sow ADG: Parity class tended to be significant: 1 vs. 2, *P* = 0.068; piglets BW at d 28 was included as covariate and was significant (*P* = 0.05; coefficient = −0.0001)

^d^Backfat loss (mm): Parity class was significant: 2 vs. 3, *P* = 0.071; litter size and piglet BW at d 28 were included as covariates and were significant (*P* = 0.020; coefficient = −0.0001)

^e^Muscle loss (mm): Parity class was significant: 1 vs. 2, *P* = 0.0243; 2 vs. 3 not significant; 1 vs. 3, *P* = 0.056; litter size at d 28 was included as covariate and was significant (*P* = 0.020; coefficient = −0.0001; *P* = 0.03; coefficient = 0.00045)

^f^Backfat loss/Muscle loss: Initial backfat was included as factor and resulted significant (*P* = 0.019; coefficient = 0.124)

### The effect of dietary supplementation with branched-chain amino acids and/or arginine on sow blood parameters

Additional file 1: Table S2 shows the effect of the supplementation of BCAAs and/or Arg on the blood haematological parameters of the sows before farrowing and during lactation. The MCHC before farrowing (d −4) was affected by Arg (*P* < 0.05); therefore, the MCHC at d −4 was included as a covariate for the analysis of the MCHC at the other time points (d 10 and d 27). A trend of a lower MCHC and a higher LYMPHO percentage was observed for the BCAAs at d 10 and d 27, respectively (*P* < 0.1). No significant effect of BCAA and/or Arg supplementation on the other haematological parameters was observed during lactation.

Table 3 shows the effect of BCAA and Arg supplementation on the concentration of Igs, hormones, urea and glucose in the serum of the sows at d −4, d 10 and d 27. No differences regarding Igs and hormones were observed among the various dietary groups in the samples of serum at d −4 when the different diets had not already been fed to the sows. At d 10, no effect of BCAA and Arg supplementation, or their interaction, was observed regarding the concentration of IgA, IgG, prolactin and insulin; regarding the concentration of IgM, Arg supplementation showed a significant effect (*P* = 0.054). However, the orthogonal contrasts between the groups supplemented with Arg and the CO group were not significant. The concentration of glucose was significantly increased by both BCAA (*P* = 0.040) and Arg (*P* = 0.020) supplementation. At d 27, no effect of BCAA and Arg, or their interaction, was observed with respect to the concentrations of IgA, IgG, IgM, insulin, glucose and urea. The concentration of prolactin was significantly increased by both BCAA (*P* = 0.035) and Arg (*P* = 0.048) supplementation while their interaction was not significant. The orthogonal contrasts showed that each individual group supplemented with the AAs (BCAA, Arg and BCAA + Arg) had a higher concentration of prolactin as compared with the CO group (BCAA vs. CO, *P* = 0.047; Arg vs. CO, *P* = 0.047; BCAA + Arg vs. CO, *P* = 0.020); furthermore, the following orthogonal contrasts were significant: CO vs. Additions (*P* = 0.021), and the BCAA and Arg groups vs. BCAA + Arg group (*P* = 0.035). In addition, the concentration of prolactin was affected by some covariates, namely initial BW (*P* = 0.042) and litter size at birth (*P* = 0.026), and tended to be affected by the parity class (*P* = 0.080).

**Table 3:** The effect of BCAA and Arg supplementation on the sow lactating diet regarding the concentration of immunoglobulins and hormones in the blood of sows

Item	Diet^a^				SEM	*P*-value		
	**CO**	**Arg**	**BCAA**	**BCAA + Arg**		**BCAA**	**Arg**	**BCAA × Arg**
Day −4								
Sows (*n*)	8	8	7	7				
IgA, mg/mL	1.9	2.29	2.34	1.69	0.74	0.643	0.702	0.449
IgG, mg/mL	12.1	16.5	17.4	22.3	4.58	0.351	0.498	0.956
IgM, mg/mL	4.23	4.77	4.51	4.01	0.52	0.690	0.458	0.297
Prolactin, ng/mL	6.58	6.62	8.9	8.21	1.66	0.291	0.985	0.796
Insulin, mU/L	21.4	26.2	27.7	47.3	12.05	0.706	0.766	0.499
Glucose, mmol/L	3.92	3.74	4.35	4.77	0.44	0.500	0.780	0.500
Urea, mmol/L	3.9	3.52	4.46	3.56	0.35	0.270	0.460	0.470
Day 10								
Sows (*n*)	8	8	7	8				
IgA, mg/mL	0.96	2.22	2.13	1.68	0.68	0.239	0.28	0.224
IgG, mg/mL	22.9	16.5	27.7	19.5	6.06	0.541	0.452	0.870
IgM, mg/mL^b^	2.12	2.62	2.31	2.23	0.2	0.490	0.054	0.160
Prolactin, ng/mL	14.1	10.2	12.2	12	1.91	0.489	0.147	0.337
Insulin, mU/L^c^	17.8	33.4	29.6	42.5	7.77	0.220	0.152	0.849
Glucose, mmol/L	3.21	4.41	4.27	4.64	0.36	0.040	0.020	0.260
Urea, mmol/L	4.48	5.03	5.11	5.63	0.41	0.270	0.340	0.970
Day 27								
Sows (*n*)	8	8	7	7				
IgA, mg/mL	3.1	2.08	2.47	1.63	0.87	0.582	0.390	0.911
IgG, mg/mL	13.3	14.1	11.6	13.5	2.09	0.525	0.770	0.758
IgM, mg/mL	1.98	2.52	2.4	2.12	0.31	0.335	0.230	0.179
Prolactin, ng/mL^d^	3.74^A^	10.98^B^	10.87^B^	23.82^B^	2.61	0.035	0.048	0.287
Insulin, mU/L	55.7	63.3	73.1	58.8	105.5	0.666	0.841	0.688
Glucose, mmol/L	3.43	4.39	4.32	4.64	0.43	0.150	0.130	0.450
Urea, mmol/L	4.19	4.41	4.53	4.40	0.48	0.630	0.750	0.720

^a^Diet: CO = group fed a standard lactating sows’ diet; Arg = group was fed the standard lactating sows’ diet plus 22.5 g/d/sow of *L*-Arg; BCAA = group was fed the standard lactating sows’ diet plus *L*-Val, *L*-ILE and *L*-Leu at 9, 4.5 and 9 g/d/sow; BCAA + Arg = group was fed the standard lactating sows’ diet plus *L*-Val, *L*-Ile, *L*-Leu and *L*-Arg at 9, 4.5, 9 and 22.5 g/d/sow

^b^IgM, mg/mL: Orthogonal contrast were not significant

^c^Insulin, mU/L: Orthogonal contrast were not significant

^d^Prolactin, ng/mL: BCAA and Arg groups vs. BCAA + Arg group, *P* = 0.034; CO vs. BCAA, Arg and BCAA + Arg groups, *P* = 0.021. Only for prolactin serum III there was a significant effect or a trend of some covariates: Initial BW: *P* = 0.042; parity class: *P* = 0.080; litter size at birth: *P* = 0.026

^A,B^Different superscripts indicate significant difference (*P* < 0.05) between groups

### The effect of dietary supplementation with branched-chain amino acids and/or arginine on the colostrum and milk composition

Table 4 reports the effect of sow dietary treatment on the colostrum and milk composition, and the concentrations of Igs, IGF-1, putrescine, spermidine and spermine. Colostrum content in total proteins, SCC, urea and the percentage of putrescine, spermidine and spermine were not affected by the BCAAs or by Arg, or by their interaction. The interaction between BCAAs and Arg significantly influenced the quantity of lactose (*P* = 0.018), and dry matter (*P* = 0.036), and the concentrations of IgA (*P* = 0.007) and IgG (*P* = 0.033), and tended to influence the quantity of fat (*P* = 0.068) in the colostrum.

**Table 4:** The effect of BCAA and Arg supplementation on the sow lactating diet regarding the proximal composition and concentration of immunoglobulins and polyamines in colostrum and milk of sows

Item	Diet^a^				SEM	*P*-value		
	**CO**	**Arg**	**BCAA**	**BCAA + Arg**		**BCAA**	**Arg**	**BCAA × Arg**
Colostrum								
Proximal analysis								
Sows (*n*)	8	8	8	8				
Proteins, g/100 mL	19.8	21.2	21.4	20	0.95	0.245	0.282	0.144
SCC, N × 1000/mL	522	418	625	883	176	0.668	0.664	0.312
Fat, %	4.18	4.47	5.69	4.08	0.50	0.036	0.682	0.068
Lactose, %^b^	3.60^A^	3.40^A^	3.19^B^	3.57^A^	0.12	0.015	0.216	0.018
Caseins, %	4.58	4.77	5.28	4.75	0.23	0.037	0.547	0.128
Urea, mg/100 mL	46.5	47	48.6	43.1	2.74	0.602	0.900	0.279
Dry matter, mg/100 mL	15.20^A^	15.80^A^	17.40^B^	15.30^A^	0.6	0.014	0.489	0.036
Immunological and physiological parameters								
Sows (*n*)	8	8	8	8				
IgA, mg/mL^c^	5.66	7.78	9.1	3.94	1.69	0.062	0.207	0.007
IgG, mg/mL^d^	31.5	35.3	44.8	26.4	8.33	0.060	0.565	0.033
IgM, mg/mL^e^	0.85	1.49	1.21	1.28	0.27	0.268	0.075	0.257
IGF-1, μg/mL	63.5	108	56.5	51.2	17.78	0.790	0.082	0.170
Biogenic amines								
Sows (*n*)	8	8	8	8				
Putrescine, nmol/mL	0.83	0.70	0.78	0.66	0.28	0.733	0.901	0.994
Spermidine, nmol/mL	11.0	10.8	11.4	10.2	1.12	0.795	0.898	0.659
Spermine, nmol/mL	4.81	5.51	10.81	4.40	3.09	0.158	0.866	0.247
Milk d 10								
Proximal analysis								
Sows (*n*)	7	6	8	6				
Proteins, g/100 mL	7.25	7.3	6.94	7.81	0.42	0.571	0.938	0.340
SCC, N × 1000/mL	590	1242	153	42	453	0.498	0.287	0.414
Fat, %	10.11	8.64	8.86	9.04	1.38	0.529	0.434	0.554
Lactose, %	5.4	5.22	5.76	5.75	0.22	0.275	0.553	0.708
Caseins, %	3.36	3.13	3.02	3.04	0.2	0.252	0.409	0.532
Urea, mg/100 mL	42.8	41.8	44.2	47.2	3.06	0.743	0.814	0.527
Dry matter, mg/100 mL	21.8	19.8	20.3	20.5	1.63	0.518	0.368	0.493
Immunological and physiological parameters								
Sows (*n*)	8	8	7	7				
IgA, mg/mL^f^	2.18	2.57	2.47	4.34	1.20	0.795	0.736	0.148
IgG, mg/mL	0.25	0.21	0.26	0.23	0.03	0.776	0.358	0.875
IgM, mg/mL^g^	0.37^A^	0.31^A^	0.49^A^	1.06^B^	0.17	0.593	0.769	0.062
IGF-1, μg/mL	50.3	33.8	21.4	38	16.73	0.183	0.498	0.325
Biogenic amines								
Sows (*n*)	8	8	7	7				
Putrescine, nmol/mL	2.28	1.64	1.66	3.79	0.94	0.600	0.616	0.139
Spermidine, nmol/mL^h^	32.3	28.9	34.6	44.8	6.50	0.794	0.719	0.315
Spermine, nmol/mL	6.57	6.07	6.16	17.39	4.44	0.946	0.940	0.221
Milk d 20								
Proximal analysis								
Sows (*n*)	6	8	6	7				
Proteins, g/100 mL	6.77	7.67	7.14	6.87	0.43	0.527	0.132	0.176
SCC, N × 1000/mL	18,530	392	899	1031	6486	0.099	0.066	0.180
Fat, %	8.38	9.05	7.71	7.91	0.68	0.559	0.498	0.734
Lactose, %	5.43	5.96	5.68	5.83	0.17	0.380	0.046	0.292
Caseins, %	3.21	3.13	2.94	2.72	0.24	0.476	0.826	0.737
Urea, mg/100 mL	36.9	42.7	45	47.3	3.25	0.140	0.221	0.599
Dry matter, mg/100 mL	20.2	20.9	19.1	19.1	0.74	0.348	0.561	0.719
Immunological and physiological parameters								
Sows (*n*)	8	8	8	7				
IgA, mg/mL^i^	0.88^A^	3.04^A^	6.06^B^	3.65^A^	1.53	0.004	0.241	0.059
IgG, mg/mL	0.31	0.2	0.27	0.43	0.07	0.687	0.287	0.069
IgM, mg/mL	0.29	0.24	0.26	0.33	0.05	0.630	0.449	0.256
IGF-1, μg/mL	53.8	74.3	14.1	31	24.78	0.241	0.517	0.938
Biogenic amines								
Sows (*n*)	8	8	8	8				
Putrescine, nmol/mL	1.43	1.26	1.27	2.06	0.51	0.828	0.823	0.365
Spermidine, nmol/mL	25.5	24.9	27.1	30.2	2.50	0.640	0.871	0.456
Spermine, nmol/mL	5.94	3.50	4.20	6.59	1.40	0.386	0.227	0.099

^a^Diet: CO = group fed a standard lactating sows’ diet; Arg = group was fed the standard lactating sows’ diet plus 22.5 g/d/sow of *L*-Arg; BCAA = group was fed the standard lactating sows’ diet plus *L*-Val, *L* -ILE and *L* -Leu at 9, 4.5 and 9 g/d/sow; BCAA + Arg = group was fed the standard lactating sows’ diet plus *L* -Val, *L*-Ile, *L* -Leu and *L* -Arg at 9, 4.5, 9 and 22.5 g/d/sow

^b^Lactose, colostrum: Parity class, *P* = 0.018; batch, *P* = 0.001

^c^IgA, mg/mL: BCAA and Arg groups vs. BCAA + Arg group, *P* = 0.020

^d^IgG, mg/mL: BCAA and Arg groups vs. BCAA + Arg group, *P* = 0.10; parity class had a significant effect *P* = 0.001

^e^IgM, mg/mL: Parity class had a significant effect: *P* = 0.04; N. piglets post fostering had a significant effect: *P* = 0.03

^f^IgA, mg/mL, milk d 10: Parity class had a significant effect *P* = 0.04

^g^IgM, mg/mL, milk d 10: BCAA and Arg groups vs. BCAA + Arg group, *P* = 0.014)

^h^Spermidine, %, milk d 10: Parity class, *P* = 0.038

^i^IgA, mg/mL, milk d 20: BCAA and Arg groups vs. BCAA + Arg group, *P* = 0.07; BCAA vs. Arg group *P* = 0.07

^A,B^Different superscripts indicate significant difference (*P* < 0.05) between groups

The orthogonal contrasts showed a significant reduction in IgA (*P* = 0.024) and a trend of reduction in IgG (*P* = 0.1) in the BCAA + Arg group as compared with the Arg and BCAA groups. The BCAAs increased the amount of fat, caseins and dry matter, and reduced the level of lactose (*P* < 0.05) in the colostrum; the orthogonal contrast also showed a significant increase in dry matter and a decrease in lactose in the BCAA group as compared with the CO group (*P* < 0.05) and a trend to a higher amount of fat and caseins in the BCAA group as compared with the CO group (*P* < 0.1). Arginine tended to increase the IgM (*P* = 0.075) and IGF-1 (*P* = 0.082) concentrations in the colostrum; however, the orthogonal contrasts were not significant.

At d 10, milk composition, IgA, IgG, IGF-1, putrescine, spermidine and spermine were not affected by either the BCAA or Arg. The IgM concentration tended to be affected by the interaction between the BCAAs and Arg (*P* = 0.062), and the orthogonal contrast showed that BCAAs + Arg had a higher concentration of IgM as compared with the BCAA and Arg groups (*P* = 0.014).

At d 20, the quantity of proteins, fat, caseins, urea and dry matter, the concentrations of IgM and IGF, and the percentages of putrescine and spermidine in the milk were not affected by either the BCAAs or Arg. The interaction between the BCAAs and Arg tended to influence the concentrations of IgA (*P* = 0.059) and IgG (*P* = 0.069), and the orthogonal contrast between the BCAA and the Control groups showed a trend to a higher concentration of IgA in the BCAA group as compared with the control group (*P* = 0.09) while the other contrasts regarding IgG were not significant. The interaction between the BCAAs and Arg tended to influence the percentage of spermine (*P* = 0.099). The BCAAs significantly increased the concentration of IgA (*P* = 0.004), and the orthogonal contrast between the BCAA group and the CO group showed a significant increase in IgA in the BCAA group (*P* = 0.01). The SCC tended to be reduced by both the BCAAs (*P* = 0.099) and Arg (*P* = 0.066). Orthogonal contrasts between the CO and the BCAA groups, and between the CO and the Arg groups showed a trend to the reduction in SCC for the BCAA and the Arg groups (*P* < 0.1). Arginine significantly increased the quantity of lactose (*P* = 0.046); however, the orthogonal contrasts between the Arg groups and the CO group were not significant.

### The effect of dietary supplementation with branched-chain amino acids and/or arginine on the faecal microbiota of sows

**Fig. 1:**
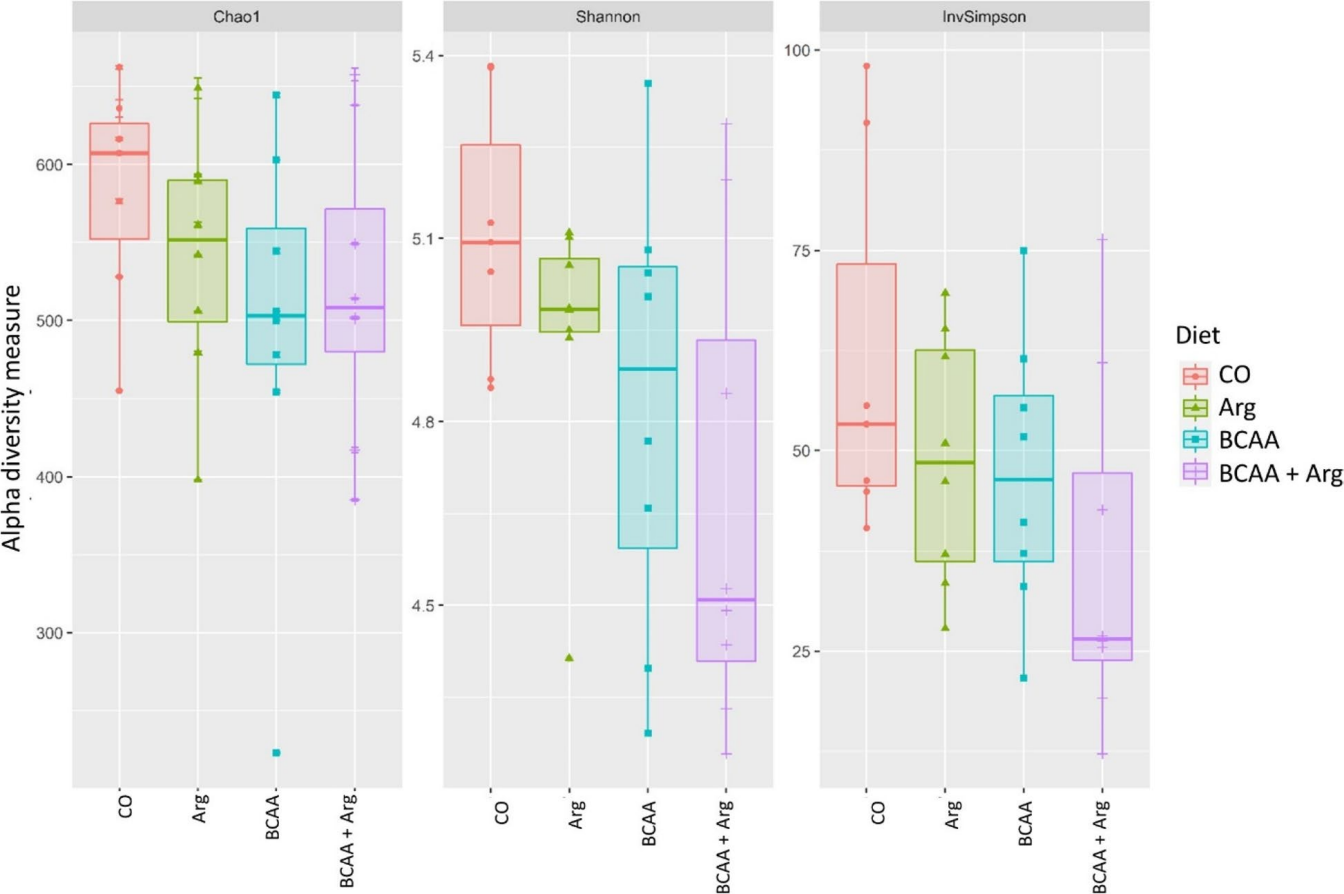
The effect of BCAAs and Arg supplementation on the Chao1, Shannon and InvSimpson index values of the sow faeces at the end of lactation. Chao1: BCAA, *P* = 0.085; Orthogonal contrast: CO vs. Additions, *P* < 0.0001; alone vs. mixed addition, *P* < 0.0001. Shannon: BCAA, *P* = 0.091; Orthogonal contrast: CO vs. Additions, *P* < 0.0001; alone vs. mixed addition, *P* < 0.0001. Diet: CO = the group fed a standard lactating sow diet; Arg = the group fed the standard lactating sow diet plus 22.5 g/d/sow of *L*-Arg; BCAA = the group fed the standard lactating sow diet plus *L-*Val, *L*-Ile and *L*-Leu at 9, 4.5 and 9 g/d/sow; BCAA + Arg = the group fed the standard lactating sow diet plus *L-*Val, *L*-Ile and *L*-Leu and *L*-Arg at 9, 4.5, 9 and 22.5 g/d/sow

**Fig. 2:**
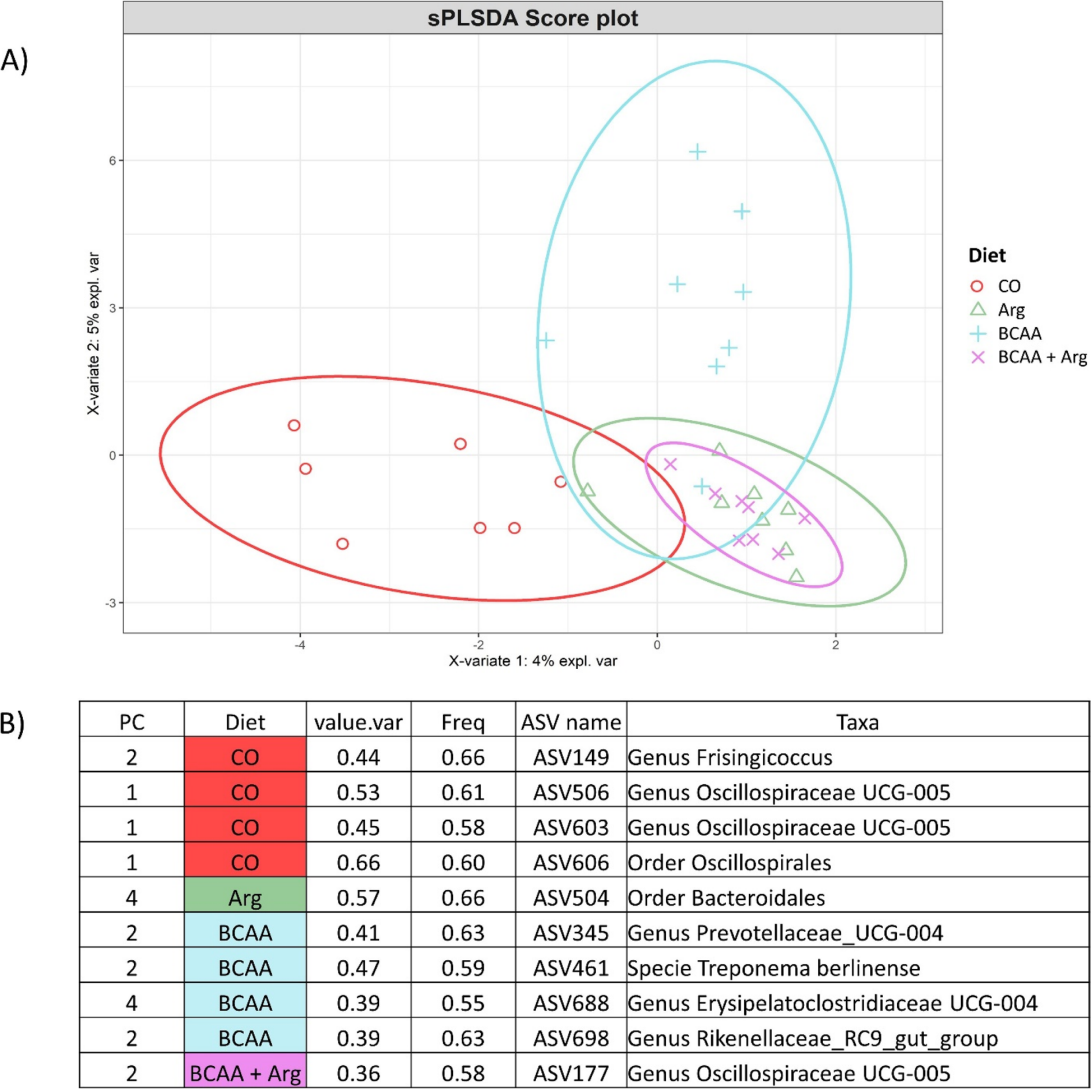
The results of the PLS-DA analysis regarding the faecal microbiota of the sows at the end of lactation. **A** Individual score plot of the samples along the first two components. **B** Table reporting the most discriminant genera per group. PC stands for the principal component which discriminates the genera; Diet: CO = the group fed a standard lactating sow diet; Arg = the group fed the standard lactating sow diet plus 22.5 g/d/sow of *L*-Arg; BCAA = the group fed the standard lactating sow diet plus *L-*Val, *L*-Ile and *L*-Leu at 9, 4.5 and 9 g/d/sow; BCAA + Arg = the group fed the standard lactating sow diet plus *L-*Val, *L*-Ile and *L*-Leu and *L*-Arg at 9, 4.5, 9 and 22.5 g/d/sow.; value.var expresses the variance explained by the single genera; Freq expresses the frequencies by which the genera were chosen among the 100 repetitions of the cross validation

A total of 1,312,970 reads were attributed to a total of 2536 ASVs distributed among samples as shown in Additional file 1: Table S3. The relative rarefaction illustrates the tendency to plateau for all the samples, suggesting that the sequencing depth was sufficient to describe the variability within the microbial communities analysed (Fig. S1). The taxonomic assignment allowed obtaining 18 phyla, 76 families and 159 genera. The alpha diversity indices of the four groups are reported in Fig. 1. The BCAA tended to reduce the Chao1, and the Shannon indices (*P* = 0.08; *P* = 0.09, respectively) while Arg, and the interaction between Arg and BCAAs, did not affect the indices. The contrasts showed that supplementation with AAs (the BCAA, Arg and BCAA + Arg groups) significantly reduced the Chao1 and Shannon indices as compared with the CO group (*P* < 0.001); furthermore, the BCAA + Arg group had lower Chao1 and Shannon indices as compared with the BCAA and Arg groups (*P* < 0.001). The InvSimpson index tended to be lower in the BCAA + Arg group as compared with the CO Group (*P* = 0.06) (Fig. 1). The beta diversity was not influenced by the BCAA and Arg supplementation, as observed by the results of the Adonis procedure (*P* > 0.1); in fact, the NMDS plot did not evidence any cluster of samples due to the diet (Fig. S2). No difference in the taxa abundancy between each BCAA group and the BCAA + Arg group as compared with the CO group was observed at the phylum, family or genera level. The comparison between the Arg group and the CO group showed a lower abundance of the Veillonellaceae family (*P*_adj._ < 0.0001; log_2_ Fold Change: 21.7) and of *Megasphaera* genera (*P*_adj._ < 0.0001; log_2_ Fold Change: 21.6) in the Arg group. To identify the discriminant taxa which belonged to the specific dietary groups, the PLS-DA was carried out, and the results are reported in Fig. 2. The Arg group was discriminated by bacteria belonging to the order Bacteroidales; the BCAA group was discriminated by bacteria belonging to the genera Prevotellaceae_UCG-004, Erysipelatoclostridiaceae UCG-004 and the Rikenellaceae_RC9_gut_group, and the specie *Treponema* *berlinense*; the BCAA + Arg group was discriminated by bacteria belonging to the genus Oscillospiraceae UCG-005, and the CO group was discriminated by bacteria belonging to the order Oscillospirales and the genera Oscillospiraceae UCG-005 and *Frisingicoccus.*

### Piglet performance and mortality

Table 5 reports the effect of sow dietary treatment on piglet performance. No effect of BCAAs was observed on piglet birth BW while Arg supplementation to the sow diet tended to improve piglet BW at birth (*P* = 0.052). The interaction between the BCAAs and Arg showed a trend (*P* = 0.08) to piglet BW at d 41. At d 41, piglets born from sows supplemented with BCAAs, Arg or BCAA + Arg were heavier than the piglets from sows fed without supplementations (CO vs. Additions, *P* = 0.03). The variability of piglet BW within the litter (CV) was tested and was shown not to be significantly affected by sow dietary treatment (data not shown).

**Table 5:** The effect of BCAA and Arg supplementation on the sow lactating diet regarding the growth performance of their piglets

Item	Piglets (*n*)	Diet^a^				SEM	*P*-value					
		**CO**	**Arg**	**BCAA**	**BCAA + Arg**		**BCAA**	**Arg**	**BCAA × Arg**	**Sow**	**Piglet BW d 0**	**Coef piglet BW d 0** ^b^
Body weight, g												
d 0^c^	866	1392	1517	1431	1510	51	0.76	0.052	0.653	0.040	-	
d 27	759	7008	7199	7147	7196	189	0.719	0.539	0.705	0.085	< .0001	2.37
d 41^d^	709	10,265	11,111	10,960	10,851	272	0.428	0.195	0.080	0.702	< .0001	3.66
Average daily gain, g/d												
d 0–7^e^	783	180	199	189	180	8.2	0.515	0.572	0.093	0.697	< .0001	0.075
d 7–14	765	198	226	222	225	12.2	0.329	0.215	0.312	0.049	< .0001	0.063
d 0–14	765	188	214	206	203	9.3	0.706	0.246	0.117	0.136	< .0001	0.072
d 14–27	758	233	219	223	234	9.1	0.782	0.901	0.176	0.225	0.003	0.03
d 0–27	759	210	217	214	217	7.2	0.73	0.484	0.79	0.081	< .0001	0.051
d 27–34	709	192	206	212	205	12.7	0.452	0.806	0.382	0.251	< .0001	0.086
d 0–34	716	206	215	213	212	6.1	0.679	0.512	0.378	0.256	< .0001	0.063
d 27–41^f^	709	213	256	249	244	13.6	0.353	0.156	0.064	0.183	< .0001	0.082
d 34–41^g^	709	239	317	295	288	18.4	0.451	0.068	0.023	0.181	< .0001	0.083
d 0–41^h^	709	211	231	227	225	60.0	0.451	0.181	0.087	0.680	0.7986	0.063

^a^Diet: CO = group fed a standard lactating sows’ diet; Arg = group was fed the standard lactating sows’ diet plus 22.5 g/d/sow of *L*-Arg; BCAA = group was fed the standard lactating sows’ diet plus *L*-Val, *L*-Ile and *L*-Leu at 9, 4.5 and 9 g/d/sow; BCAA + Arg = group was fed the standard lactating sows’ diet plus *L*-Val, *L*-Ile, *L*-Leu and *L*-Arg at 9, 4.5, 9 and 22.5 g/d/sow

^b^Covariate coefficient

^c^Body weight, d 0; parity class was significant (*P* = 0.008). Coefficients for the class of parity: 1, + 225 g; 2, + 61; 3, 0

^d^Body weight, d 41: Orthogonal contrast: CO vs. BCAA, Arg and BCAA + Arg groups, *P* = 0.026

^e^Average daily gain d 0—d –7: Orthogonal contrasts were not significant

^f^Average daily gain d 27–41: Orthogonal contrast: CO vs. BCAA, Arg and BCAA + Arg groups, *P* = 0.015

^g^Average daily gain d 34–41: Orthogonal contrast: CO vs. BCAA, Arg and BCAA + Arg groups, *P* = 0.005

^h^Average daily gain d 0–41: Orthogonal contrast: CO vs. BCAA, Arg and BCAA + Arg groups, *P* = 0.027

Piglets ADG from d 0 to d 7 displayed a trend to the interaction between the BCAAs and Arg (*P* = 0.093); however, none of the contrasts was significant. The ADGs for the periods d 7–14, d 0–14, d 14–27, d 0–27, d 27–34, and d 0–34 were not affected by either the BCAAs, Arg or their interaction. A trend to a higher ADG in the periods d 27–41 (*P* = 0.064) and d 0–41 (*P* = 0.087) was observed for BCAA and Arg interaction. The ADG for the periods d 27–41 and at d 0–41 was higher in piglets from the sows supplemented with BCAAs, Arg or BCAA + Arg than in piglets from sows without supplementation (CO vs. Additions, d 27–41, *P* = 0.015; d 0–41: *P* = 0.027). The ADG in the period d 34–41 was significantly influenced by the BCAA and Arg interaction (*P* = 0.023), and a trend to a higher value of the ADG was observed for Arg in the same period (*P* = 0.068). Orthogonal contrast showed that the ADG at d 34–41 of the BCAA, Arg or BCAA + Arg groups was, on average, higher than that of the piglets from sows without supplementation (*P* = 0.005).

**Fig. 3:**
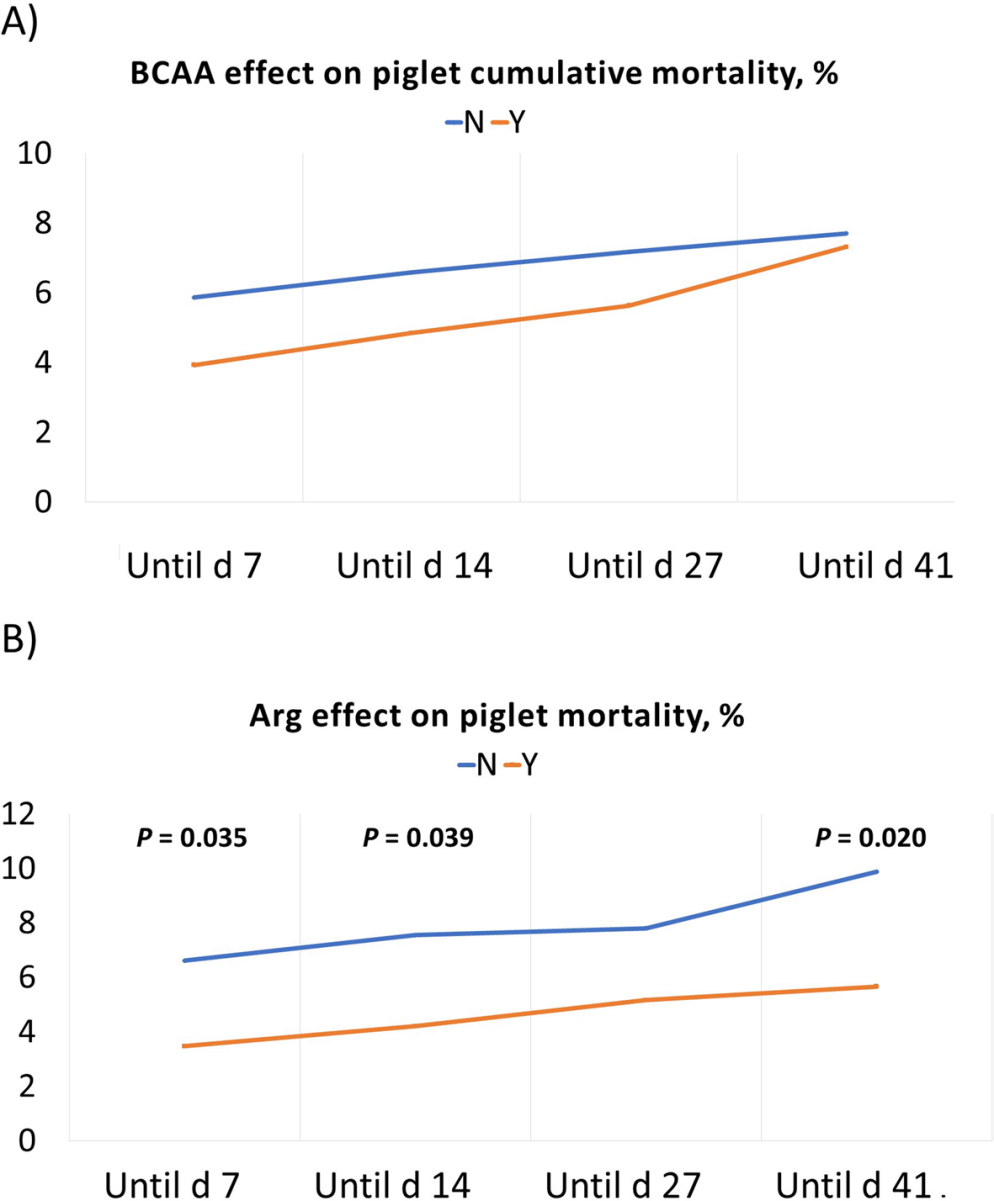
The effect of BCAAs and Arg supplementation on the sow lactation diet regarding piglet mortality until d 41 after birth. **A** The effect of BCAA supplementation; **B** The effect of Arg supplementation

Figure 3 shows the effect of BCAA (Fig. 3A) and Arg (Fig. 3B) supplementation to the sow lactation diet on piglet mortality. The BCAA supplementation did not affect piglet mortality while the Arg supplementation to the sow diet reduced the piglet mortality calculated until d 7 (*P* = 0.035), until d 14 (*P* = 0.039) and until d 41 (*P* = 0.020).

### Piglet blood parameters

Table 6 shows the effect of BCAA and/or Arg supplementation on the haematological parameters, urea, glucose and Igs concentration in the serum of the piglets at d 10 and d 27. Overall, the values obtained for all the parameters were within the normal range, confirming that all the piglets included in the study were healthy [38].

**Table 6:** The effect of BCAA and Arg supplementation on the sow lactating diet regarding the haematological blood parameters, glucose urea and immunoglobulins concentration of their pigets

Item^a^	Piglets (*n*)	Diet^b^				SEM	*P*-value		
		**CO**	**Arg**	**BCAA**	**Arg + BCAA**		**BCAA**	**Arg**	**BCAA × Arg**
Day 10									
RBC, 10^6^/µL	62	5.43	5.39	5.21	5.24	0.18	0.212	0.982	0.849
HGB, g/dL	62	11.89	11.71	11.51	11.41	0.29	0.127	0.561	0.868
HCT, %	62	35.79	35.11	34.34	34.04	1.15	0.157	0.601	0.839
MCV, fL	62	66.38	65.16	66.06	64.97	1.31	0.802	0.293	0.954
MCH, pg	62	22.11	21.73	22.19	21.84	0.54	0.821	0.425	0.967
MCHC, g/dL	62	33.26	33.35	33.61	33.66	0.46	0.366	0.849	0.958
PLT, 10^3^/μL	62	847.3	688.2	760.2	798.1	96.2	0.876	0.435	0.206
WBC, 10^3^/µL	62	5.81	4.87	4.94	4.98	0.63	0.437	0.397	0.342
NEUTRO, %	62	49.12	56.5	53.02	48.48	4.81	0.571	0.713	0.130
LYMPHO, %	62	39.98	31.84	36.12	39.85	5.41	0.608	0.606	0.173
MONO, %	62	7.31	9.62	8.12	9.06	1.57	0.920	0.198	0.584
EOSI, %	62	1.00	0.86	1.59	1.54	0.52	0.126	0.833	0.909
BASO, %	62	0.4	0.65	0.63	0.5	0.26	0.850	0.771	0.357
Glucose, mmol/L	55	7.06	6.87	7.13	7.1	0.45	0.659	0.768	0.820
Urea, mmol/L	55	1.84	2.17	2.11	2.46	0.26	0.196	0.154	0.980
IgA, mg/mL^c^	49	0.59	1.04	0.62	0.3	0.25	0.095	0.760	0.084
IgG, mg/mL	49	7.28	6.46	5.07	4.87	2.40	0.300	0.793	0.868
IgM, mg/mL	50	0.25	0.32	0.25	0.31	0.06	0.986	0.197	0.948
Day 27									
RBC, 10^6^/µL	60	6.39	6.61	6.51	6.6	0.14	0.688	0.302	0.65
HGB, g/dL	60	11.48	12.11	12.03	12.25	0.25	0.179	0.121	0.423
HCT, %	60	34.64	35.2	35.45	36.37	0.85	0.239	0.404	0.827
MCV, fL	60	54.12	53.42	54.2	55.26	0.78	0.338	0.868	0.391
MCH, pg	60	17.93	18.38	18.39	18.6	0.35	0.314	0.357	0.723
MCHC, g/dL	60	33.18	34.35	33.9	33.66	0.43	0.968	0.302	0.108
PLT, 10^3^/μL	60	610.19	771.74	825.47	811.74	74.13	0.085	0.340	0.238
WBC, 10^3^/µL	60	10.3	9.69	9.48	8.32	1.22	0.358	0.488	0.823
NEUTRO, %	60	34.58	42.33	42.11	39.36	3.56	0.504	0.498	0.137
LYMPHO, %^d^	60	56.12	36.94	48.8	52.07	4.43	0.359	0.086	0.012
MONO, %	60	5.9	10.44	4.91	7.77	1.49	0.213	0.025	0.572
EOSI, %	60	1.59	2.19	1.7	2.67	0.54	0.565	0.160	0.730
BASO, %	60	0.57	1.27	0.87	0.74	0.29	0.674	0.346	0.162
Glucose, mmol/L	63	8.21	7.49	7.56	6.83	0.47	0.155	0.136	0.985
Urea, mmol/L	63	1.62	1.66	1.87	1.95	0.26	0.310	0.831	0.931
IgA, mg/mL	55	0.28	0.4	0.35	0.5	0.07	0.229	0.095	0.851
IgG, mg/mL	55	5.13	5.11	6.11	5.49	0.56	0.257	0.652	0.632
IgM, mg/mL	55	0.51	0.56	0.61	0.52	0.07	0.673	0.741	0.357

^a^RBC: red blood cell count; HGB: haemoglobin; HCT: haematocrit; MCV: mean corpuscular volume; MCH: mean corpuscular haemoglobin; MCHC: mean corpuscular haemoglobin concentration; PLT: platelet count; WBC: white blood cell count; NEUTRO: neutrophil; LYMPHO: lymphocyte; MONO: monocyte; EOSI: eosinophil; BASO: basophil

^b^Diet: CO = group fed a standard lactating sows’ diet; Arg = group was fed the standard lactating sows’ diet plus 22.5 g/d/sow of *L*-Arg; BCAA = group was fed the standard lactating sows’ diet plus *L*-Val, *L*-Ile and *L*-Leu at 9, 4.5 and 9 g/d/sow; BCAA + Arg = group was fed the standard lactating sows’ diet plus *L*-Val, *L*-Ile, *L*-Leu and *L*-Arg at 9, 4.5, 9 and 22.5 g/d/sow

^c^IgA, mg/mL, d 10: BCAA and Arg groups vs. BCAA + Arg group, *P* = 0.055

^d^LYMPHO, % Orthogonal contrast: CO vs. BCAA, Arg and BCAA + Arg groups, *P* = 0.0450; BCAA and Arg groups vs. BCAA + Arg group, *P* = 0.096; BCAA vs. Arg group, *P* = 0.060

At d 10, neither BCAA nor Arg supplementation, nor their interaction, influenced the haematological parameters, or the concentrations of glucose, urea, IgM and IgG. A trend to a higher concentration of IgA was observed for the interaction between the BCAAs and Arg (*P* = 0.084).

At d 27, neither BCAA nor Arg supplementation, or their interaction, influenced the levels of RBC, HGB, HCT, MCV, MCH, MCHC, and WBC, the percentage of NEUTRO, EOSI and BASO, and the concentrations of urea, glucose, IgG and IgM. A trend to a higher level of PLT was found for BCAA supplementation (*P* = 0.085). The percentage of LYMPHO was significantly influenced by the interaction between BCAA and Arg (*P* = 0.012), and tended to be reduced by Arg supplementation (*P* = 0.086). The orthogonal contrast for the percentage of LYMPHO showed a significant effect of BCAA or Arg supplementations as compared with the CO group (CO vs. Additions, *P* = 0.045) while a trend was found for other contrasts (the BCAA and Arg groups vs. the BCAA + Arg group, *P* = 0.096; the BCAA vs. Arg group, *P* = 0.060). The Arg supplementation significantly increased the percentage of MONO (*P* = 0.025). A trend to a higher concentration of IgA was observed with the Arg supplementation (*P* = 0.095).

### Piglet mucosal gene expression

**Fig. 4:**
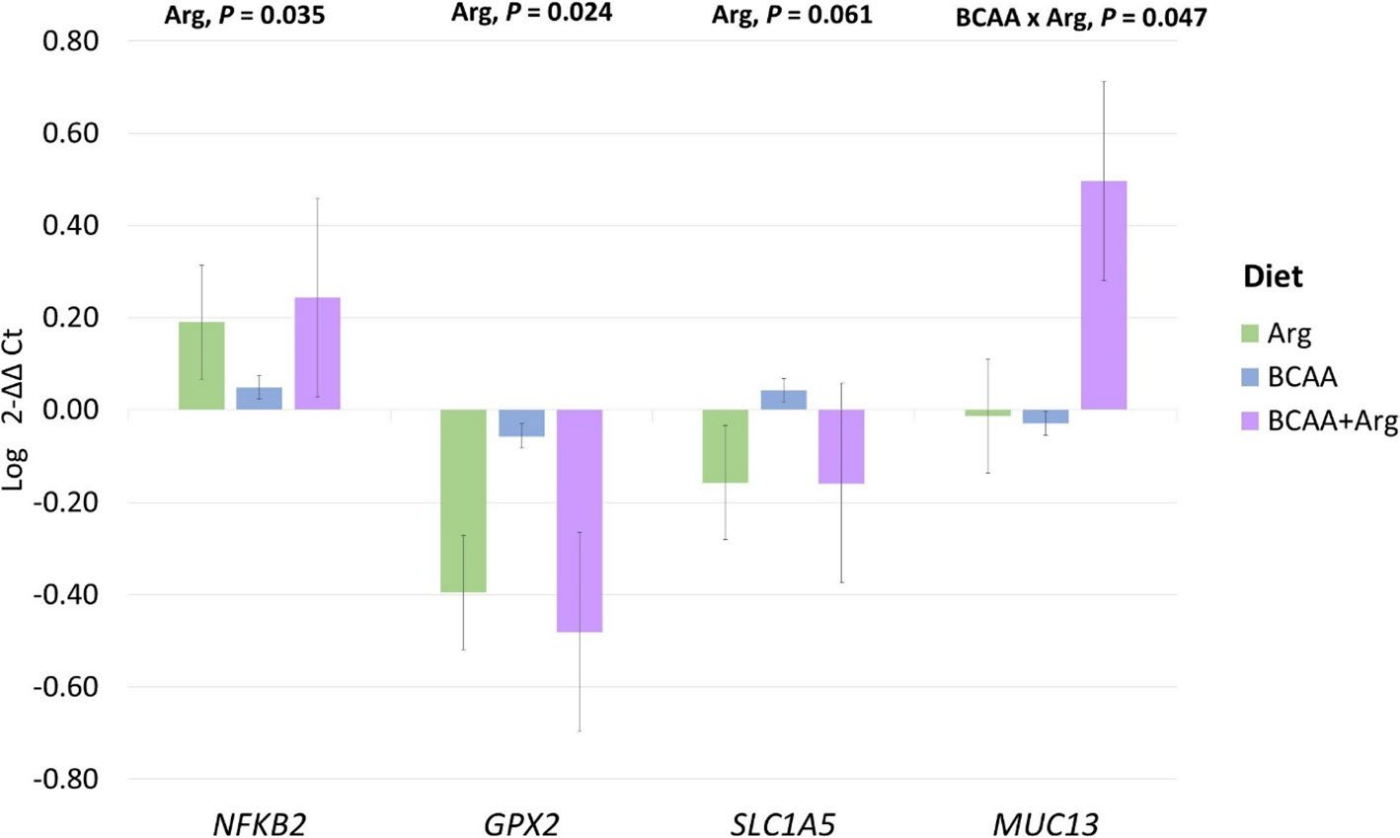
The effect of BCAAs and Arg supplementation on sows regarding the jejunal gene expression of their piglets. Diet: Arg = the group fed the standard lactating sow diet plus 22.5 g/d/sow of *L*-Arg; BCAA = the group fed the standard lactating sow diet plus *L-*Val, *L*-Ile and *L*-Leu at 9, 4.5 and 9 g/d/sow; BCAA + Arg = the group fed the standard lactating sow diet plus *L-*Val, *L*-Ile and *L*-Leu and *L*-Arg at 9, 4.5, 9 and 22.5 g/d/sow

Neither BCAA nor Arg supplementation, or their interaction, influenced the expression of *ZO*-1, *OCL*, *MyD88*, *CLAUD3*, *CLAUD4*, *PIGR*, *BCAT2 SLC7A9*, *SLC38A2*, *SCL6A19* and *ODC1* in the jejunal mucosa of the piglets. The Arg supplementation to the sow diet increased the expression of *NFKB2* (*P* = 0.035), reduced the expression of *GPX-2* (*P* = 0.024) and tended to reduce the expression of *SLC1A5* (*P* = 0.061) in the jejunal mucosa of their offspring. The interaction between BCAA and Arg supplementation to the sow diet affected the expression of *MUC13* (*P* = 0.047) which was highest in the BCAA + ARG group (Fig. 4).

## Discussion

The aim of the present study was to investigate the effect of BCAAs and Arg, alone or in combination, and above the nutritional requirement, on the physiological parameters, microbial profile and performance of lactating sows and their offspring. The absence of any effect of BCAAs and Arg on the body variation of the sows, in terms of ADG, backfat and muscle loss, observed in the present study agreed with previous studies in which BCAAs [39–42] and Arg [8, 43] were added to the feed of the sows at higher levels than the standard NRC requirements [23]. This confirmed the validity of the NRC requirement.

Regarding the blood parameters of the sows, BCAAs supplementation tended to reduce the MCHC, and significantly increased the glucose concentrations in the blood at d 10 of lactation. Although the MCHC values of all the sows in the study were in line with the normal value, the decrease in the MCHC coupled with no effect on the HGB suggested a reduction in the concentration of HGB in the red blood cells. Although no information regarding the effect of BCAA supplementation on haematological parameters has been found regarding sows, studies on humans have suggested an effect of dietary BCAAs on the haematological parameters, especially regarding HGB and the iron metabolism, due to the activation of the mechanistic target of rapamycin complex 1 (mTORC1) [44, 45]. Increased glucose in the blood from BCAA supplementation could be associated with the increased transportation of glucose to the mammary gland, where it can be used for the production of lactose (via galactose conversion), rather than to the muscle; in fact, in lactating sows, the transport of glucose to muscle is not a priority for protein synthesis [46]. In general, insulin favours the porcine mammary uptake of glucose and mammary protein synthesis [47]; however, studies ruminants have shown that glucose uptake from the MG could be insulin-independent via glucose transporters, particularly type 1 (*GLUT-1*) [48, 49]. In agreement with that, at this phase of lactation, the insulin in the blood was not affected in the present study. In addition, higher blood glucose could also be associated with a reduction in the BCAA catabolism; in fact, Li et al. showed that a higher level of BCAAs could affect the glucose metabolism and increase the glucose concentration in the blood [50].

At the end of the lactation period (d 27), BCAAs supplementation tended to increase the percentage of lymphocytes in the peripheral blood and significantly increased the concentration of prolactin. The BCAAs, especially Leu, are known to be involved in the immune response mechanisms. Lymphocytes express BCAA transaminase and branched-chain 2-oxoacid dehydrogenase for BCAA degradation; therefore, greater BCAAs availability may have increased lymphocyte mitogenesis [51, 52]. However, no previous study has been found which is in agreement with the present results. In addition, few studies have evaluated the effect of BCAAs on the prolactin level of sow blood; however, in agreement with the present study, Gao et al. [53] observed an increase in sow blood prolactin in response to Val supplementation during lactation. On the contrary, an intravenous supplementation of 18% of the daily intake of Ile, Leu and Val did not affect prolactin concentrations from day 7 to day 10 of lactation [54]. However, in the same trial, de Ridder and co-authors [54] evaluated the effect of BCAAs on the prolactin concentration during the initial lactation period while, in the present study, the effect was observed at the end of lactation (d 27) when the milk production was more intensive for the sows due to the increasing demand for milk by the piglets. Thus, the results cannot be completely comparable. In the present study, however, it was not possible to measure the milk yield, and the results of the milk composition at d 10 and d 20 did not show any effect regarding BCAAs supplementation.

Conversely, the BCAA supplementation affected the colostrum composition, increasing fat and caseins, and reducing lactose. In general, BCAAs are considered particularly important for lipid synthesis in the MG since they play a key role in the inhibition of branched chain ketoacid dehydrogenase (BCKD) kinase, the overexpression of which activates de novo lipogenesis in the liver [55]. In agreement with the present results, Ma et al. [56] observed an increase in fat in the colostrum of sows fed a diet enriched with BCAAs from day 107 of gestation regardless of the level of fat in the diet. The BCAAs are also considered signalling molecules able to modulate milk protein synthesis. For instance, Val supplementation during sow gestation significantly increased the colostrum protein [57], and both Val and Leu promoted total and cell-specific proteins (beta-lactoglobulins) by means of the activation of the mTOR pathway in porcine, bovine and murine in vitro studies [57, 58]. In the current study, a significant difference in the total protein content was not observed; however, the casein level was increased by the BCAAs. No studies involving sows have reported any effect of BCAAs on the casein percentage; however, it can be assumed that this could be an indirect effect of BCAAs on milk protein production. On the contrary, the reduction of colostrum lactose by BCAAs could be indirectly associated with the increase in solid concentration, fat and caseins in the colostrum since lactose is generally recognised to be an osmotic factor [13].

The lack of a BCAA effect on the milk composition at days 10 and 20 of lactation observed in the present study could have been related to the functionality of the MG itself. In fact, as reported by Kim et al. [59, 60], the MG itself undergoes accelerated proliferation between days 75 and 114 of gestation, and from day 0 to day 14 of lactation while it decreases thereafter. Thus, since the effect of BCAAs can be targeted to the mammary epithelial cells, which could explain the effect of BCAAs on colostrum composition and, conversely, the lack of effect in mature milk composition observed in this study. In addition to affecting the colostrum composition, BCAAs supplementation tended to increase the concentrations of IgA and IgG in the colostrum, and the concentration of IgA in the milk at day 20. It should be noted that these effects were also significant for the interaction between the BCAAs and Arg.

Arginine supplementation increased the concentration of glucose and tended to increase the concentration of IgM in the blood of the sows at d 10 of lactation. Arginine is known to affect multiple metabolic pathways including glucose synthesis [61, 62]. It has been shown that an elevated dietary amount of Arg can be used to produce creatinine which, when converted to creatine, can improve glucose tolerance [63, 64]; this mechanism may explain the present results. Furthermore, it has been shown that dietary Arg can favour the uptake of glucose from the blood to produce muscle, and at the same time, it reduces the transport of glucose to the adipose tissue (glucose transporter type 4 expression) fattening pigs [65]. However, as previously mentioned, lactating sows do not have a net deposition of muscle proteins, while they lose muscle proteins, which would explain the higher circulation of glucose observed in the present study.

How Arg acts on the immune system is not entirely clear. The literature suggests that dietary Arg supplementation can enhance the immune function in various models of immunological challenges [66] by acting as a substrate for protein synthesis or as a precursor of polyamines or nitric oxide. The latter are important for sustaining cell proliferation and for the stimulation of T cell receptor expression, T-lymphocyte proliferation, and B cell development [66]. In agreement with the present study, the study of Che et al.[67] suggested that Arg supplementation during gestation improved sow serum Igs [67]. Interestingly, in this study, the colostrum of the sows supplemented with Arg was also richer in IgM. The present results partially agreed with the results reported by Nuntapaitoon et al. [21] in whose study the Arg supplementation during gestation improved colostrum Igs (IgG). Overall, the present results suggested that Arg supplementation to sows could result in increased protection of the guts of the newborn piglets by means of richer initial passive immunity.

Arginine is also recognised as stimulating the secretion of key anabolic hormones, including insulin and GH, in pigs [10], and prolactin in dairy cattle [11]. In agreement with Chew et al. [11], an increase in prolactin concentration in the blood at d 27 in sows supplemented with Arg was observed in the present study. Previous results regarding the effect of Arg on blood prolactin are contradictory. In fact, Zhu et al. [43] observed an increase in blood prolactin at d 14 and d 21 of lactation with supplementation of 1.0% *L-*Arg-HCl. On the contrary, Pérez Laspiur et al. [7] observed an increase in prolactin concentration in the blood of sows undergoing dietary supplementation with Arg (1.73% or 1.34% as compared with the standard (0.96%)) only under heat stress conditions while no effect was observed at d 7, d 14, and d 21 of lactation under normal environmental conditions. Prolactin is involved in a multiplicity of actions, and it is of particular importance in gestating and lactating animals. In sows, prolactin in late gestation plays a key role in the lactogenic and galactopoietic processes, and it is recognised as a major factor in milk yield [68, 69]. In the present study, it was not possible to measure the milk yield; however, in agreement with the increase in blood prolactin, an increase in lactose in milk at d 20 was observed.

In the present study, no modification of blood insulin was observed, and blood GH was not analysed. However, the concentration of insulin-like growth factor‐I, which is released from the liver in response to GH [70], was analysed in the colostrum and milk. In agreement with the study of Krogh et al. [13], in the present study, a trend of higher Insulin‐like growth factor‐I in the colostrum was observed. In infants, IGF-1 plays a significant role by stimulating the AA uptake in muscle for protein deposition, of glucose uptake to promote energy storage and cell growth, and proliferation via the mTORC1 pathway [71]. Furthermore, IGF-1 can contribute to promoting intestinal tissue growth and functional maturation in newborn animals by increasing the cell proliferation in the intestinal crypts [72] and the villus height [73].

The increase in both IGF-1 and IgM in the colostrum can partially explain the reduced pre- and post-weaning mortality rate which was observed in the present study in the piglets of the sows supplemented with Arg. Lower post-weaning mortality of the offspring of Arg-fed sows has previously been reported by Hines et al. [74]. In agreement with the lower mortality rate, the ratio of monocytes to lymphocytes changed in the blood serum of piglets at d 27, being more in favour of monocytes for piglets suckling from sows having Arg-supplemented diets. This was mainly due to an increase in the monocyte counts in these piglets (data not shown). Monocytes are important for the health of pre-weaning piglets as they can differentiate into macrophages and dendritic cells, and thus be able to capture antigens, produce stimulatory cytokines and display phagocytosis activity [75], all of which are very important at the time of weaning.

The results obtained for jejunal gene expression at weaning showed that piglets suckling from sows having Arg-supplemented diets had a higher expression of the *NFKB2* gene and a reduced expression of *GPX-2*, both of which are considered markers for immune response and inflammation in the gastrointestinal mucosa [76, 77]. Previous studies have reported that GPX-2 can exert an inhibitory effect on NF-κB signalling and its target gene expression [78, 79]; furthermore, a connection between the *NFKB2* expression and the production of monocyte-derived macrophages has been suggested [80]. Therefore, the results of the present study suggested that feeding the sows with Arg could modulate the intestinal homeostasis of their piglets, increasing the intestinal expression of *NFKB2*, together with a downregulation of the expression of *GPX-2*, and resulting in an increase in the blood monocytes of their offspring. However, the complete mode of action still needs to be elucidated. Overall, at least for the piglets in the Arg groups, this may have contributed to better performance after weaning (second week) as compared with the control group, observed in the present study. Partially in agreement with the present findings, the studies of Hines et al. [74], Zhu et al. [43] and Mateo et al. [9] have reported an improved pre-weaning ADG in the piglets of the sows fed a lactation diet supplemented with Arg. Unfortunately, these studies did not follow the piglets during the post-weaning period. However, Oksbjerg et al. [14] reported an improved ADG from weaning to 140 kg of the pigs reared by sows supplemented with Arg during late gestation and lactation. On the other hand, Dallanora et al. [81] and Krogh et al. [13] reported that Arg supplementation during late gestation and lactation did not improve piglet performance.

However, in the present study, the growth performance of the piglets was influenced by the interaction between Arg and the BCAAs more than by the “individual” AA additions. This result suggested a positive synergy between these AAs which is not easy to explain. The higher ADG of the piglets could have been associated with the effect that the BCAAs and Arg had on the milk immunoglobulins (higher IgM in the milk at d 10, higher IgA in the milk at d 20) as it is known that better immunological protection in early life would have favoured additional development of the piglets [82]. In addition, BCAA + Arg supplementation tended to prompt the concentration of spermine in milk at d 20, which could also contribute to explaining the improvement in piglet performance as spermine can promote the maturation of the mucosa, maintaining intestinal integrity, thereby improving epithelial restitution and barrier function after stress injury [83, 84]. The mechanisms by which BCAAs + Arg improved milk spermine need to be clarified. As a general hypothesis, which needs to be supported by specific studies, it is possible that the synergy between the BCAAs and Arg is at the level of the interaction between insulin and the BCAAs (primarily leucine) on the MTOR-RPS6K-RPS6-EIF4EBP1 signal transduction pathway. In addition, it can be hypothesised that the elevated level of AAs in this group produced a modification of the sow microbiota in the small intestine resulting in a greater production of spermine which was then transported via blood to the milk. To support this hypothesis, the study of Hu et al. showed a significant modification in the faecal microbiota of the pigs fed a supplementation of Arg and Leu [85]. Moreover, it has been suggested that AAs can regulate the gut microbiota composition and activity and, as a consequence, the host metabolism [4]. The present study showed that the continuous supplementation of free Arg and BCAAs to the sows during the entire lactation period did not notably affect the faecal microbial profile. This was not surprising if one considered that dietary free amino acids were expected to be absorbed in the upper intestinal tracts. Nevertheless, the AA supplementation could have affected the general rate of absorption of the AAs by the host and the AAs availability for the microbes, starting from the small intestine [86]. In fact, a reduction in the alpha diversity (Chao and Shannon indices) was observed in the sows fed additional free AAs; in particular, the reduction was more pronounced with the supplementation of the BCAAs and that of both the BCAAs and Arg. Previous studies have suggested that a high alpha diversity of gut microbiota would be beneficial as it favours greater plasticity in response to perturbations, while a low gut microbial diversity has been associated with higher adiposity, insulin resistance, dyslipidaemia and inflammation in humans [87]. In swine, it has been observed that highly prolific sows have a lower gut microbial richness than sows with a low productive capacity during gestation, and a higher gut microbial richness than low productive capacity sows after farrowing [88]. In general, there is no univocal interpretation of these indices of microbial diversity as their meaning can vary depending on species and conditions. Moreover, the results of the present study suggested that specific taxa, which are common in sow intestinal microbiota, could be influenced by either Arg or BCAA supplementation. Arginine supplementation promoted the settlement of bacteria belonging to the order Bacteroidales and reduced the settlement of bacteria belonging to the Veillonellaceae family which agreed with the results obtained by Luise et al. [15] in which Arg supplementation significantly reduced the Veillonellaceae in gestating sows [15]. In addition, in the present study, Arg supplementation significantly reduced the abundance of the *Megasphaera* genus (belonging to the Veillonellaceae family). Bacteria of this genus are typically the dominant users of lactate in the digestive tracts [89]. The BCAAs supplementation promoted the settlement of bacteria belonging to the genus *Erysipelatoclostridiaceae UCG-004*, and to *Prevotellaceae_UCG-004*, and Rikenellaceae_RC9_gut_group, which are commonly found in the gut microbial community of pigs [90]. *Prevotella* is generally recognised for its capacity of using both simple and complex dietary saccharides [91, 92]; however, a previous study on mice has suggested a positive correlation between *Prevotella* abundancy and BCAA circulation in the blood [93]. In agreement with that, BCAAs supplementation in mice significantly increased the abundance of *Prevotella* in the colon [94]. In fact, specific species of *Prevotella*, namely *Prevotella ruminicola*, are known for their relevant role in protein digestion and AA absorption in the mammalian digestive tract [95]. This result agreed with the present study and increased interest in the role which *Prevotella* may have in the interplay in the digestive tract of pigs. Likewise, *Prevotella,* and the Rikenellaceae_RC9_gut_group have been found to increase with the increasing level of dietary crude protein in adult pigs [96] and with BCAAs supplementation in mice [94]. Therefore, the results observed in the present study were supported by previous evidence and they highlighted the relationship between dietary BCAAs and the abundance of *Prevotella* and Rikenellaceae*.* The family Erysipelatoclostridiaceae has recently been proposed as a new genome-based bacterial taxon of the previously known phylogenetic *Clostridium* VIII species by Park et al. [97]. Bacteria belonging to Clostridia are known to be involved in AA fermentation and its subsequent absorption [98]; however additional relationships between Erysipelatoclostridiaceae and BCAA have yet to be determined.

## Conclusions

In conclusion, the present study showed that BCAAs and Arg supplementation during the entire lactation period can benefit sow productive performance in terms of piglet ADG, immune competence and survivability, via modulation of the metabolism, colostrum and milk composition and the intestinal microbiota of the sows. In detail, the supplementation of both BCAAs and Arg alone increased the glucose and prolactin in sows blood, the BCAAs increased the IgA and IgM concentrations in the milk while Arg increased the sow IgM in the serum and colostrum. These results supported the knowledge that BCAAs and Arg were involved in several physiological pathways and could play a key role in the physiology of lactating sows. Furthermore, the synergistic effect observed in this study between these AAs and noticeable by a trend in increased Igs and spermine in the milk and in the improvement of the performance of the piglets merits additional research. It remains to be verified in future studies whether the effect of BCAA and Arg supplementation on the health and performance of piglets could also be related to a modulation of the AA composition of the colostrum and milk.

## Supplementary Information

**Additional file 1: Table S1.** List of target genes and TaqMan assay Id for gene expression analysis of the jejunum. **Table S2.** The effect of BCAA and Arg supplementation on the sow’s haematological blood parameters. **Table S3.** Per sample information regarding sequencing depth (reads abundances), ASVs abundances (Observed) and alpha diversity indices (Chao, Shannon and InvSimpson). **Fig. S1.** Rarefaction curve of fecal samples of sows resulted by sequencing of V3–V4 regions with MiSeq platform (Illumina Inc., San Diego, CA, USA). Diet: Control = the group fed a standard lactating sow diet; Arg = the group fed the standard lactating sow diet plus 22.5 g/d/sow of *L*-Arg; BCAA = the group fed the standard lactating sow diet plus *L*-Val, *L*-Ile and *L*-Leu at 9, 4.5 and 9 g/d/sow; BCAA + Arg = the group fed the standard lactating sow diet plus *L*-Val, *L*-Ile and *L*-Leu and *L*-Arg at 9, 4.5, 9 and 22.5 g/d/sow. **Fig. S2.** NMDS plot on Bray Curtis distance matrix on faecal samples of sows fed a lactation diet supplemented with arginine and/or BCAA. Diet: Control = the group fed a standard lactating sow diet; Arg = the group fed the standard lactating sow diet plus 22.5 g/d/sow of *L*-Arg; BCAA = the group fed the standard lactating sow diet plus *L*-Val, *L*-Ile and *L*-Leu at 9, 4.5 and 9 g/d/sow; BCAA + Arg = the group fed the standard lactating sow diet plus *L*-Val, *L*-Ile and *L*-Leu and *L*-Arg at 9, 4.5, 9 and 22.5 g/d/sow.

## Data Availability

The data regarding the microbiota generated and analysed during the current study are available in the NCBI Sequence Read Archive (SRA) repository under accession number PRJNA859270. The other datasets used and/or analysed during the current study are available from the corresponding author on reasonable request.

## Abbreviations

AAs: Amino acids
ADG: Average daily gain
Arg: Arginine
ASV: Amplicon sequence variant
BA: Born alive piglets
BASO: Basophil
BCAAs: Branched-chain amino acids
BCAT2: Branched chain amino acid transaminase 2
BCKD: Branched chain ketoacid dehydrogenase
BW: Body weight
CLAUD3: Claudin-3
CLAUD4: Claudin-4
CO: Control
Ct: Cycle threshold
ELISA: Enzyme-linked immunosorbent assay
EOSI: Eosinophil
GH: Growth hormone
GLM: General linear model
GLUT-1: Glucose transporters type 1
GPX-2: Cell surface associated glutathione peroxidase 2
HCT: Haematocrit
HGB: Haemoglobin
IgA: Immunoglobulin A
IGF-1: Insulin growth factors
IgG: Immunoglobulins G
IgM: Immunoglobulin M
Igs: Immunoglobulins
*L*-Ile: *L*-Isoleucine
*L*-Leu: *L*-Leucine
LPT: Platelet count
*L-*Val: *L-*Valine
LYMPHO: Lymphocyte
MCH: Mean corpuscular haemoglobin
MCHC: Mean corpuscular haemoglobin concentration
MCV: Mean corpuscular volume
MG: Mammary gland
MONO: Monocyte
mTORC1: Mechanistic target of rapamycin complex 1
MUC13: Mucin 13
MyD88: Innate immune signal transduction adaptor
NEUTRO: Neutrophil
NFKB2: Nuclear factor kappa B subunit 2
NMDS: Non-metric multidimensional scaling
NRC: National Research Council
OCLN: Occludin
ODC1: Ornithine decarboxylase 1
PIGR: Polymeric immunoglobulin receptor
REML: Restricted maximum likelihood
SB: Stillborn
SCC: Count of somatic cells
SEM: Standard error of the mean
SLC1A5: Solute carrier family 1 member 5
SLC38A2: Solute carrier family 38 member 2
SLC6A19: Solute carrier family 6 (neutral amino acid transporter), member 19
SLC7A9: Solute carrier family 7 member 9
sPLS-DA: Sparse partial least squares discriminant analysis
WBC: White blood cell count
Zo-1: Tight junction protein 1
ΔCt: Delta cycle threshold
