# Supplementary material for: Productive and physiological implications of top-dress addition of branched-chain amino acids and arginine on lactating sows and offspring
Source: J Anim Sci Biotechnol. 2023 Mar 7;14:40. doi: 10.1186/s40104-022-00819-8 (PMC9990366; doi:10.1186/s40104-022-00819-8)
Supplement: Supplementary file 1 — Additional file 1: Table S1. List of target genes and TaqMan assay Id for gene expression analysis of the jejunum. Table S2. The effect of BCAA and Arg supplementation on the sow’s haematological blood parameters. Table S3. Per sample information regarding sequencing depth (reads abundances), ASVs abundances (Observed) and alpha diversity indices (Chao, Shannon and InvSimpson). Fig. S1. Rarefaction curve of fecal samples of sows resulted by sequencing of V3–V4 regions with MiSeq platform (Illumina Inc., San Diego, CA, USA). Diet: Control = the group fed a standard lactating sow diet; Arg = the group fed the standard lactating sow diet plus 22.5 g/d/sow of L-Arg; BCAA = the group fed the standard lactating sow diet plus L-Val, L-Ile and L-Leu at 9, 4.5 and 9 g/d/sow; BCAA + Arg = the group fed the standard lactating sow diet plus L-Val, L-Ile and L-Leu and L-Arg at 9, 4.5, 9 and 22.5 g/d/sow. Fig. S2. NMDS plot on Bray Curtis distance matrix on faecal samples of sows fed a lactation diet supplemented with arginine and/or BCAA. Diet: Control = the group fed a standard lactating sow diet; Arg = the group fed the standard lactating sow diet plus 22.5 g/d/sow of L-Arg; BCAA = the group fed the standard lactating sow diet plus L-Val, L-Ile and L-Leu at 9, 4.5 and 9 g/d/sow; BCAA + Arg = the group fed the standard lactating sow diet plus L-Val, L-Ile and L-Leu and L-Arg at 9, 4.5, 9 and 22.5 g/d/sow. [file 40104_2022_819_MOESM1_ESM.docx]

**Table S1** List of target genes and TaqMan assay Id for gene expression analysis of the jejunum

| **Target gene** | **Complete name** | **TaqMan assey Id** |
| --- | --- | --- |
|  |  |  |
|  |  |  |
| *MyD88* | Innate Immune Signal Transduction Adaptor | Ss03389125_m1 |
| *NFKB2* | Nuclear Factor Kappa B Subunit 2 | Ss06883741_g1 |
| *OCLN* | Occludin | Ss03377507_u1 |
| *ZO-1* | Tight Junction Protein 1 | Ss03373514_m1 |
| *MUC13* | Mucin 13, Cell Surface Associated | Ss03386544_u1 |
| *GPX2* | Glutathione Peroxidase 2 | Ss03387478_u1 |
| *CLAUD4* | Claudin-4 | Ss03375006_u1 |
| *CLAUD3* | Claudin-3 | Ss04328819_g1 |
| *PIGR* | Polymeric Immunoglobulin Receptor | Ss03394221_m1 |
| *BCAT2* | Branched Chain Amino Acid Transaminase 2 | Ss06438438_m1 |
| *ODC1* | Ornithine Decarboxylase 1 | Ss03390110_m1 |
| *SLC6A19* | Solute Carrier Family 6 Member 19 | Ss03376931_u1 |
| *SLC7A9* | Solute Carrier Family 7 Member 9 | Ss03386787_u1 |
| *SLC1A5* | Solute Carrier Family 1 Member 5 | Ss03376929_u1 |
| *SLC38A2* | Solute Carrier Family 38 Member 2 | Ss06886416_m1 |
| *HMBS* | Hydroxymethylbilane Synthase | Ss03388782_g1 |

**Table S2** The Effect of BCAA and Arg supplementation on the sow’s haematological blood parameters

| **Item**^a^ | **Diet^b^** | | | | **SEM** | ***P* - value** | | | | | |
| --- | --- | --- | --- | --- | --- | --- | --- | --- | --- | --- | --- |
|  | **CO** | **Arg** | **BCAA** | **BCAA + Arg** |  | **BCAA** | **Arg** | **BCAA**  **x Arg** | **Batch** | **initial**  **BW** | **Parity**  **class** |
| Day -4 | | | | | | | | | | | |
| RBC 10^6^/µL | 6.12 | 6.06 | 5.77 | 5.67 | 0.27 | 0.37 | 0.87 | 0.95 | 0.46 | 0.88 | 0.08 |
| HGB g/dL | 12.9 | 12.6 | 12.2 | 12.1 | 0.52 | 0.35 | 0.63 | 0.83 | 0.6 | 0.98 | 0.04 |
| HCT, % | 36.2 | 36.4 | 34.3 | 34.1 | 1.52 | 0.39 | 0.95 | 0.9 | 0.43 | 0.74 | 0.05 |
| MCV, fL | 59.2 | 60.1 | 59.5 | 60.2 | 0.77 | 0.81 | 0.47 | 0.93 | 0.18 | 0.11 | 0.57 |
| MCH, pg | 21.2 | 20.8 | 21.2 | 21.4 | 0.32 | 0.90 | 0.43 | 0.43 | 0.06 | 0.75 | 0.17 |
| MCHC g/dL^c^ | 35.7 | 34.6 | 35.7 | 35.5 | 0.23 | 0.85 | <0.005 | 0.06 | 0.01 | 0.02 | 0.03 |
| PLT 10^3^/μL | 173 | 132 | 143 | 152 | 23.65 | 0.37 | 0.26 | 0.33 | 0.46 | 0.03 | 0.55 |
| WBC 10^3^/µL | 9.79 | 9.58 | 11.11 | 9.98 | 0.58 | 0.13 | 0.81 | 0.47 | 0.28 | 0.08 | 0.79 |
| NEUTRO, % | 61.5 | 61.7 | 59.8 | 59.8 | 2.09 | 0.59 | 0.95 | 0.95 | 0.33 | 0.41 | 0.71 |
| LYMPHO, % | 23.3 | 22.4 | 25.3 | 23.7 | 2.32 | 0.51 | 0.79 | 0.9 | 0.77 | 0.86 | 0.64 |
| MONO, % | 9.6 | 10.59 | 8.62 | 10.28 | 1.05 | 0.52 | 0.54 | 0.77 | 0.49 | 0.38 | 0.64 |
| EOSI, % | 5 | 4.29 | 5.09 | 5.36 | 0.91 | 0.94 | 0.61 | 0.63 | 0.08 | 0.73 | 0.32 |
| BASO, % | 0.62 | 1.08 | 1.14 | 0.89 | 0.21 | 0.10 | 0.17 | 0.14 | 0.33 | 0.59 | 0.56 |
| Day 10 | | | | | | | | | | | |
| RBC 10^6^/µL | 5.19 | 4.95 | 5.37 | 5.36 | 0.29 | 0.65 | 0.53 | 0.69 | 0.37 | 0.81 | 0.47 |
| HGB g/dL | 11 | 10.7 | 11.4 | 11.4 | 0.54 | 0.54 | 0.67 | 0.8 | 0.4 | 0.78 | 0.43 |
| HCT, % | 30.6 | 29.7 | 32 | 31.7 | 1.57 | 0.50 | 0.66 | 0.84 | 0.39 | 0.71 | 0.42 |
| MCV, fL | 58.9 | 60.1 | 59.8 | 59.6 | 0.78 | 0.40 | 0.24 | 0.35 | 0.71 | 0.68 | 0.66 |
| MCH, pg | 21.1 | 21.6 | 21.3 | 21.4 | 0.32 | 0.57 | 0.26 | 0.53 | 0.51 | 0.8 | 0.70 |
| MCHC g/dL | 35.7 | 36.1 | 35.1 | 36 | 0.24 | 0.06 | 0.34 | 0.37 | 0.05 | 0.28 | 0.49 |
| PLT 10^3^/uL | 239 | 280 | 221 | 264 | 20.68 | 0.53 | 0.13 | 0.98 | 0.17 | 0.52 | 0.19 |
| WBC 10^3^/µL | 13.3 | 13.7 | 13.3 | 12.7 | 1.08 | 0.98 | 0.82 | 0.66 | 0.51 | 0.41 | 0.48 |
| NEUTRO, % | 68.1 | 71.1 | 68.7 | 68.4 | 2.52 | 0.86 | 0.36 | 0.5 | 0.24 | 0.6 | 0.19 |
| LYMPHO, % | 21.2 | 18.6 | 21.6 | 20.8 | 2.48 | 0.89 | 0.43 | 0.72 | 0.34 | 0.79 | 0.37 |
| MONO, % | 7.09 | 7.4 | 6.13 | 7.19 | 0.7 | 0.31 | 0.73 | 0.59 | 0.38 | 0.2 | 0.19 |
| EOSI, % | 2.84 | 2.21 | 2.58 | 2.86 | 0.58 | 0.74 | 0.40 | 0.42 | 0.67 | 0.01 | 0.01 |
| BASO, % | 0.79 | 0.63 | 0.95 | 0.75 | 0.23 | 0.59 | 0.62 | 0.91 | 0.03 | 0.66 | 0.50 |
| Day 27 | | | | | | | | | | | |
| RBC 10^6^/µL | 5.47 | 5.14 | 5.29 | 5.29 | 0.22 | 0.57 | 0.29 | 0.44 | 0.94 | 0.69 | 0.54 |
| HGB g/dL | 11.6 | 11 | 11.3 | 11.2 | 0.40 | 0.62 | 0.25 | 0.53 | 0.61 | 0.49 | 0.67 |
| HCT, % | 31.9 | 30.6 | 31.2 | 30.4 | 1.09 | 0.62 | 0.43 | 0.86 | 0.65 | 0.43 | 0.69 |
| MCV, fL | 58.3 | 59.8 | 59.1 | 57.5 | 0.84 | 0.53 | 0.24 | 0.08 | 0.26 | 0.4 | 0.28 |
| MCH, pg | 21.3 | 21.4 | 21.5 | 21.1 | 0.29 | 0.77 | 0.85 | 0.46 | 0.28 | 0.45 | 0.31 |
| MCHC g/dL | 36.3 | 36.4 | 36.1 | 36.5 | 0.33 | 0.69 | 0.88 | 0.70 | 0.09 | 0.48 | 0.37 |
| PLT 10^3^/uL | 248 | 287 | 251 | 271 | 28.63 | 0.95 | 0.34 | 0.74 | 0.18 | 0.91 | 0.21 |
| WBC 10^3^/µL | 14.4 | 15.5 | 12.3 | 13.9 | 1.43 | 0.30 | 0.60 | 0.85 | 0.02 | 0.43 | 0.47 |
| NEUTRO, % | 68.3 | 69.4 | 60.2 | 65.8 | 3.44 | 0.12 | 0.82 | 0.50 | 0.41 | 0.05 | 0.02 |
| LYMPHO, % | 18.4 | 19.3 | 26 | 21.6 | 2.84 | 0.07 | 0.82 | 0.34 | 0.14 | 0.02 | 0.03 |
| MONO, % | 7.95 | 7.3 | 7.89 | 7.66 | 1.05 | 0.97 | 0.66 | 0.84 | 0.54 | 0.22 | 0.22 |
| EOSI, % | 4.75 | 3.09 | 5.2 | 4.33 | 0.91 | 0.74 | 0.21 | 0.66 | 0.01 | 0.27 | 0.44 |
| BASO, % | 0.64 | 0.91 | 0.7 | 0.6 | 0.17 | 0.80 | 0.28 | 0.28 | 0.34 | 0.10 | 0.61 |

^a^ RBC: red blood cell count; HGB: haemoglobin; HCT: haematocrit; MCV: mean corpuscular volume; MCH: mean corpuscular haemoglobin; MCHC: mean corpuscular haemoglobin concentration; PLT: platelet count; WBC: white blood cell count; NEUTRO: neutrophil; LYMPHO: lymphocyte; MONO: monocyte; EOSI: eosinophil; BASO: basophil

^b^ Diet: CO = the group fed a standard lactating sow diet; Arg = the group fed the standard lactating sow diet plus 22.5 g/d/sowof *L*-Arg; BCAA = the group fed the standard lactating sow diet plus *L-*Val, *L*-Ile and *L*-Leu at 9, 4.5 and 9 g/d/sow; BCAA + Arg = the group fed the standard lactating sow diet plus *L-*Val, *L*-Ile and *L*-Leu and *L*-Arg at 9, 4.5, 9 and 22.5 g/d/sow

^c^ The initial level of MCHC (Serum I) was included as covariant the statistical model for MCHC in Serum II and Serum III

**Table S3** Per sample information regarding sequencing depth (reads abundances), ASVs abundances (Observed) and alpha diversity indices (Chao, Shannon and InvSimpson)

| **Sample_ID** | **Sow** | **Diet^a^** | **BCAA** | **Arg** | **Depth** | **Observed** | **Chao1** | **Shannon** | **InvSimpson** |
| --- | --- | --- | --- | --- | --- | --- | --- | --- | --- |
| 723781F689218 | 467 | Arg | no | yes | 40370 | 398 | 398.00 | 4.41 | 27.84 |
| 723782F689219 | 368 | Arg | no | yes | 41328 | 542 | 542.00 | 5.11 | 69.66 |
| 723783F689220 | 952 | CO | no | no | 58997 | 607 | 607.33 | 5.05 | 55.61 |
| 723784F689221 | 807 | BCAA | yes | no | 37443 | 544 | 544.50 | 5.01 | 51.68 |
| 723785F689222 | 698 | CO | no | no | 46776 | 631 | 636.00 | 5.13 | 46.28 |
| 723786F689223 | 530 | Arg | no | yes | 50253 | 593 | 593.17 | 4.94 | 37.09 |
| 723787F689224 | 750 | BCAA+Arg | yes | yes | 41970 | 501 | 501.00 | 4.53 | 25.48 |
| 723788F689225 | 465 | BCAA | yes | no | 32743 | 506 | 506.00 | 5.04 | 55.34 |
| 723789F689226 | 476 | CO | no | no | 35406 | 576 | 576.50 | 5.38 | 98.02 |
| 723790F689227 | 1017 | Arg | no | yes | 37361 | 479 | 479.33 | 5.10 | 65.16 |
| 723791F689228 | 391 | BCAA | yes | no | 34846 | 454 | 454.33 | 4.66 | 33.03 |
| 723793F689230 | 1037 | BCAA | yes | no | 42332 | 644 | 644.50 | 5.35 | 74.98 |
| 723794F689231 | 945 | BCAA+Arg | yes | yes | 61524 | 502 | 502.17 | 4.26 | 19.10 |
| 723795F689232 | 662 | BCAA | yes | no | 54708 | 477 | 478.00 | 4.29 | 21.65 |
| 723796F689233 | 364 | BCAA+Arg | yes | yes | 47760 | 549 | 549.25 | 4.33 | 12.20 |
| 723797F689234 | 373 | Arg | no | yes | 47089 | 589 | 589.00 | 5.06 | 61.70 |
| 723798F689235 | 799 | BCAA+Arg | yes | yes | 51128 | 638 | 638.00 | 5.20 | 60.97 |
| 723799F689236 | 844 | Arg | no | yes | 42179 | 560 | 561.20 | 4.98 | 46.15 |
| 723800F689237 | 158 | BCAA+Arg | yes | yes | 52754 | 654 | 657.75 | 5.29 | 76.39 |
| 723801F689238 | 359 | BCAA+Arg | yes | yes | 30470 | 416 | 417.00 | 4.44 | 26.23 |
| 723802F689239 | 252 | BCAA+Arg | yes | yes | 38538 | 514 | 514.17 | 4.85 | 42.65 |
| 723803F689240 | 617 | BCAA+Arg | yes | yes | 30553 | 385 | 385.20 | 4.49 | 26.89 |
| 723804F689241 | 642 | CO | no | no | 42303 | 528 | 528.00 | 4.87 | 40.36 |
| 723805F689242 | 473 | BCAA | yes | no | 17812 | 223 | 223.00 | 4.40 | 37.22 |
| 723806F689243 | 853 | BCAA | yes | no | 45294 | 500 | 500.00 | 4.77 | 41.10 |
| 723807F689244 | 253 | Arg | no | yes | 36316 | 506 | 506.00 | 4.95 | 50.90 |
| 723808F689245 | 382 | CO | no | no | 31293 | 455 | 455.00 | 4.86 | 44.92 |
| 723809F689246 | 243 | BCAA | yes | no | 48938 | 603 | 603.00 | 5.08 | 61.44 |
| 723810F689247 | 833 | Arg | no | yes | 52620 | 642 | 649.00 | 4.99 | 33.48 |
| 723811F689248 | 823 | CO | no | no | 55379 | 662 | 662.33 | 5.38 | 90.91 |
| 723812F689249 | 276 | CO | no | no | 46614 | 616 | 616.33 | 5.09 | 53.28 |

^a^ Diet: CO = the group fed a standard lactating sow diet; Arg = the group fed the standard lactating sow diet plus 22.5 g/d/sow of *L*-Arg; BCAA = the group fed the standard lactating sow diet plus *L-*Val, *L*-Ile and *L*-Leu at 9, 4.5 and 9 g/d/sow; BCAA + Arg = the group fed the standard lactating sow diet plus *L-*Val, *L*-Ile and *L*-Leu and *L*-Arg at 9, 4.5, 9 and 22.5 g/d/sow


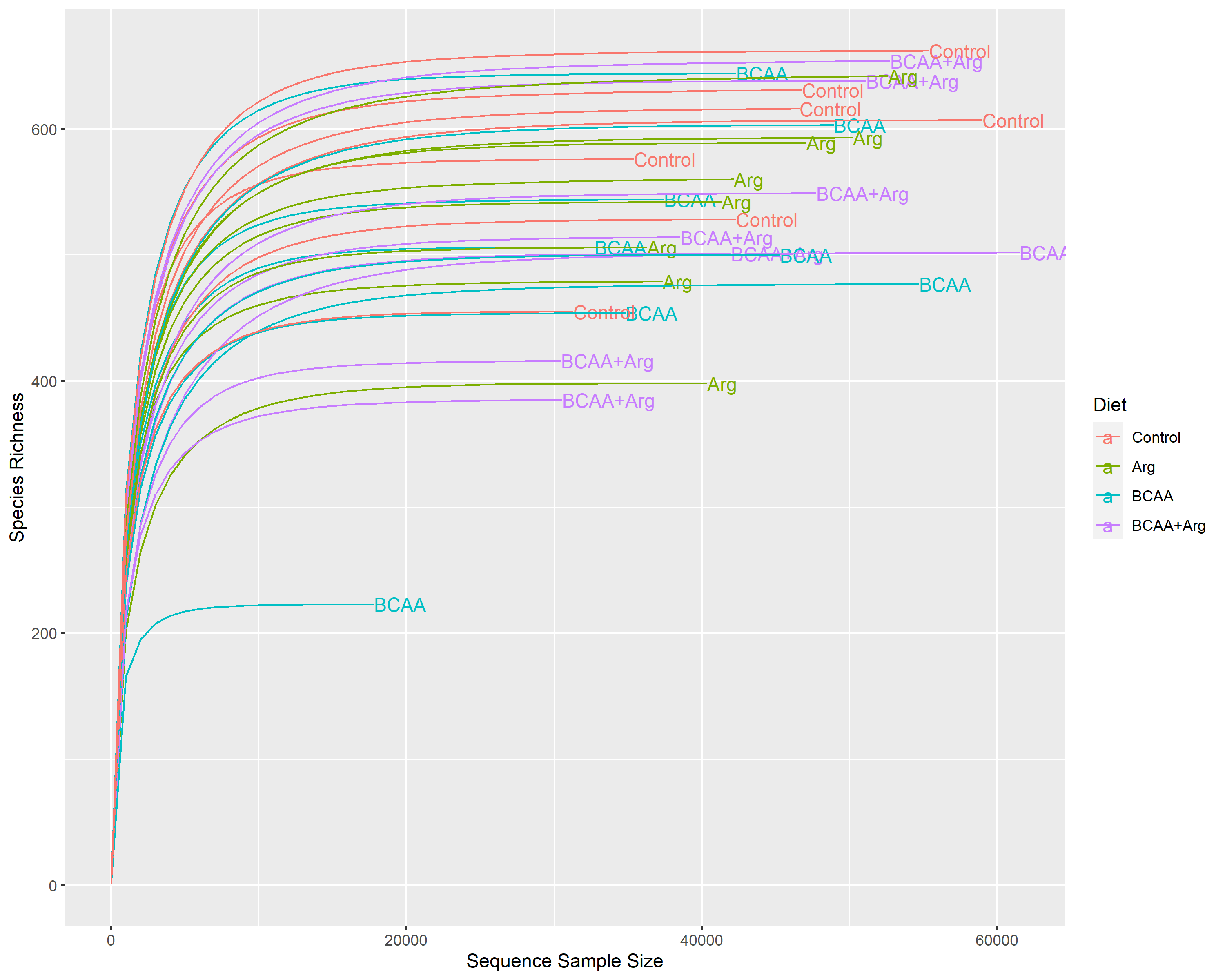


**Fig. S1** Rarefaction curve of fecal samples of sows resulted by sequencing of V3–V4 regions with MiSeq platform (Illumina Inc., San Diego, CA, USA). Diet: Control = the group fed a standard lactating sow diet; Arg = the group fed the standard lactating sow diet plus 22.5 g/d/sow of *L*-Arg; BCAA = the group fed the standard lactating sow diet plus *L-*Val, *L*-Ile and *L*-Leu at 9, 4.5 and 9 g/d/sow; BCAA + Arg = the group fed the standard lactating sow diet plus *L-*Val, *L*-Ile and *L*-Leu and *L*-Arg at 9, 4.5, 9 and 22.5 g/d/sow


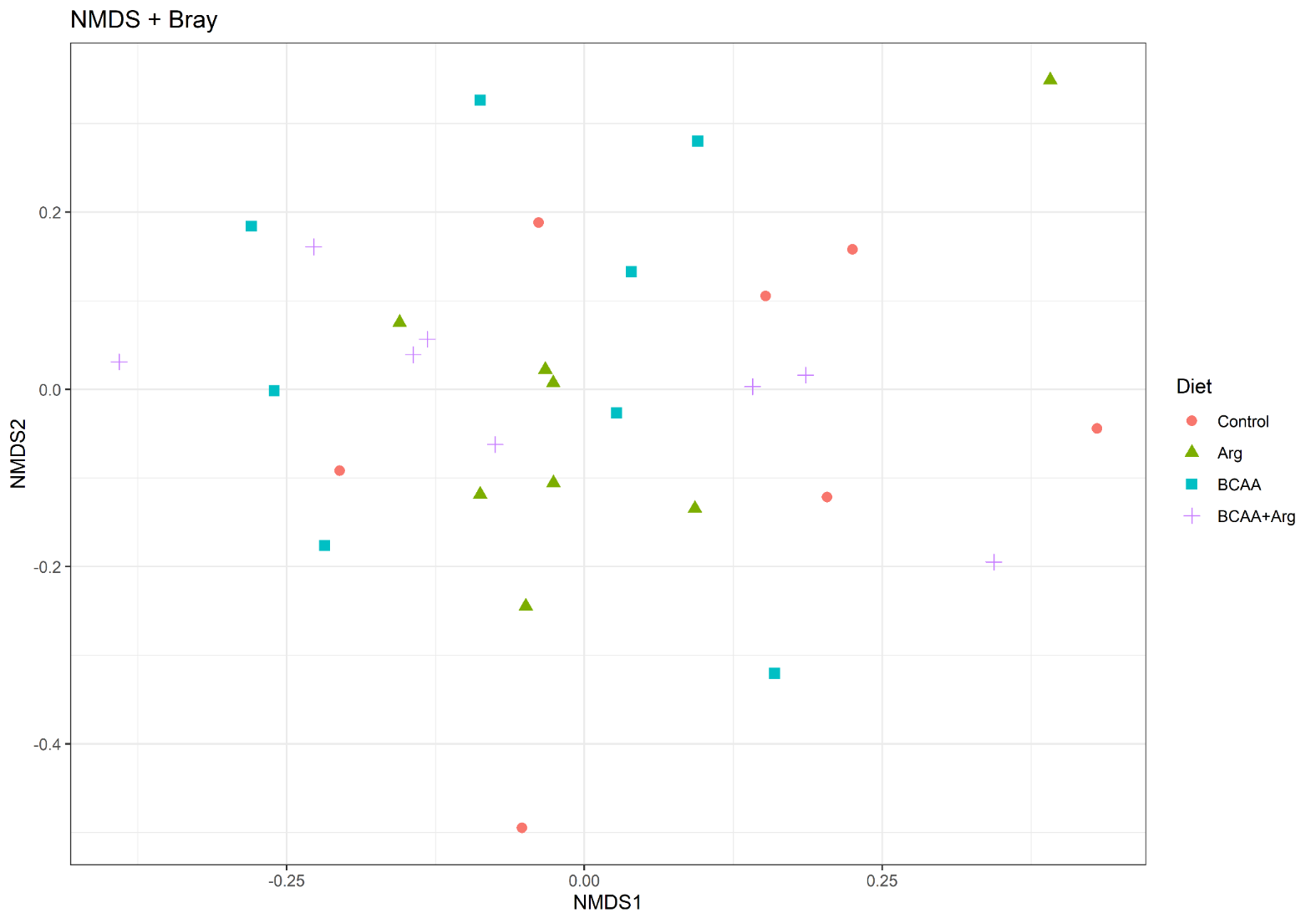


**Fig. S2** NMDS plot on Bray Curtis distance matrix on faecal samples of sows fed a lactation diet supplemented with arginine and/or BCAA. Diet^:^ Control = the group fed a standard lactating sow diet; Arg = the group fed the standard lactating sow diet plus 22.5 g/d/sow of *L*-Arg; BCAA = the group fed the standard lactating sow diet plus *L-*Val, *L*-Ile and *L*-Leu at 9, 4.5 and 9 g/d/sow; BCAA + Arg = the group fed the standard lactating sow diet plus *L-*Val, *L*-Ile and *L*-Leu and *L*-Arg at 9, 4.5, 9 and 22.5 g/d/sow
